# TRIM24 is an insulin-responsive regulator of P-bodies

**DOI:** 10.1038/s41467-022-31735-0

**Published:** 2022-07-08

**Authors:** Wen Wei, Qiaoli Chen, Minjun Liu, Yang Sheng, Qian OuYang, Weikuan Feng, Xinyu Yang, Longfei Ding, Shu Su, Jingzi Zhang, Lei Fang, Antonio Vidal-Puig, Hong-Yu Wang, Shuai Chen

**Affiliations:** 1 State Key Laboratory of Pharmaceutical Biotechnology, Department of Endocrinology, Nanjing Drum Tower Hospital, The Affiliated Hospital of Nanjing University Medical School, Model Animal Research Center, Nanjing University, Nanjing, 210061 China; 2 MOE Key Laboratory of Model Animal for Disease Study, Model Animal Research Center, School of Medicine, Nanjing University, Nanjing, 210061 China; 3 Jiangsu Key Laboratory of Molecular Medicine, Model Animal Research Center, School of Medicine, Nanjing University, Nanjing, 210061 China; 4 School of Medicine, Nanjing University, Nanjing, 210061 China; 5 TVP Lab, WT/MRC Institute of Metabolic Science, MRC Metabolic Diseases Unit - Metabolic Research Laboratories, University of Cambridge, Cambridge, UK; 6 Cambridge University Nanjing Centre of Technology and Innovation, Jiangbei Area, Nanjing, China

**Keywords:** Insulin signalling, Fat metabolism, Kinases, RNA decay, Non-alcoholic steatohepatitis

## Abstract

Insulin is a potent inducer of mRNA transcription and translation, contributing to metabolic regulation. Insulin has also been suggested to regulate mRNA stability through the processing body (P-body) molecular machinery. However, whether and how insulin regulates mRNA stability via P-bodies is not clear. Here we show that the E3-ligase TRIM24 is a critical factor linking insulin signalling to P-bodies. Upon insulin stimulation, protein kinase B (PKB, also known as Akt) phosphorylates TRIM24 and stimulates its shuttling from the nucleus into the cytoplasm. TRIM24 interacts with several critical components of P-bodies in the cytoplasm, promoting their polyubiquitylation, which consequently stabilises *Pparγ* mRNA. Inactivation of TRIM24 E3-ligase activity or prevention of its phosphorylation via knockin mutations in mice promotes hepatic *Pparγ* degradation via P-bodies. Consequently, both knockin mutations alleviate hepatosteatosis in mice fed on a high-fat diet. Our results demonstrate the critical role of TRIM24 in linking insulin signalling to P-bodies and have therapeutic implications for the treatment of hepatosteatosis.

## Abstract (web summary)

Insulin promotes hepatic lipogenesis, though underlying regulation remains unclear. Here the authors show that insulin translocates TRIM24 from the nucleus into cytosolic P-bodies to stabilise hepatic Pparγ mRNA, and that inactivation of TRIM24 promotes Pparγ degradation and alleviates hepatosteatosis.

## Introduction

After a meal, insulin elicits several metabolic responses to control nutrient surge in the body, contributing to maintaining metabolic health. Conversely, insulin resistance impairs metabolic homoeostasis and leads to the development of metabolic diseases, typically associated with obesity, such as type 2 diabetes and nonalcoholic fatty liver disease^1^. Despite intensive studies in the past few decades, the molecular mechanisms linking insulin action and resistance with those pathologies are still not fully understood.

Insulin binds to its receptor and activates the receptor kinase to elicit signal transduction through the insulin receptor substrate (IRS)–PI3-kinase (PI-3K)–protein kinase B (PKB, also known as Akt) pathway^2^. PKB phosphorylates diverse substrates that bridge insulin signalling with distinct physiological outcomes, including regulation of gene expression. For instance, in response to insulin, PKB phosphorylates a transcription factor named forkhead box protein O1 (FoxO1), promoting its nuclear exclusion^3^. FoxO1 governs transcription of *G6pase* that encodes the key gluconeogenic enzyme glucose 6-phosphatase, and FoxO1 phosphorylation by PKB mediates insulin-induced inhibition of hepatic gluconeogenesis^4^. Insulin also exerts transcriptional control of hepatic lipogenesis through composite regulation involving multiple transcription factors such as upstream stimulatory factor (USF), liver X receptor (LXR), carbohydrate response element binding protein (ChREBP) and sterol regulatory element binding protein-1c (SREBP-1c)^5^. Besides controlling transcription, insulin is also a potent inducer of global mRNA translation. Through phosphorylation by PKB, insulin inactivates tuberin/TSC2 and thereby activates the mechanistic target of rapamycin (mTOR), a master regulator of mRNA translation,^6^ which enhances protein synthesis by phosphorylating p70S6 kinase and 4E-BP1^7^.

Besides associated with ribosomes translationally-active mRNAs, cells also contain mRNAs that undergo translational repression. Translationally-repressed mRNAs can aggregate into cytoplasmic messenger ribonucleoprotein (mRNP) granules that are membrane-less organelles referred to as processing bodies (P-bodies) and stress granules^8^. P-bodies harbour multiple proteins with diverse functions to dynamically regulate translationally repressed mRNAs for storage or degradation^9,10^. Decay of mRNAs in P-bodies involves their deadenylation and decapping through coordinated actions of the deadenylation complex Ccr4-Not, the Sm-like-1-7 (LSM1-7) complex, the decapping enzyme Dcp1/Dcp2, the decapping regulators such as the enhancer of decapping protein-3 and 4 (EDC3 and EDC4), and the 5′-to-3′ exoribonuclease Xrn1^11^. P-bodies also link the microRNA (miRNA) pathway to the decay of a subset of miRNA targets by recruiting the Argonaute proteins (AGOs) that interact with the GW182 protein, a key component of P-bodies^12^. Recent evidence suggests that insulin signalling might be involved in the regulation of P-bodies^13,14^. In *Drosophila*, somatic insulin signalling regulates the organisation of P-bodies in germline cells^13^. In hepatocytes, insulin represses the translation of apolipoprotein B (ApoB) mRNA by promoting its intracellular traffic into P-bodies^14^. P-bodies may have a bi-directional relationship with insulin signalling. Deficiency of LSM1 protein, a key component of P-bodies, impairs insulin/IGF-1 signalling in *Caenorhabditis elegans*^15^. However, the biological significance and molecular mechanisms of insulin in the regulation of P-bodies remain unclear.

Tripartite motif-containing 24 (TRIM24), also known as transcriptional intermediary factor 1α (TIF1α), is a multi-domain containing protein^16^. An LXXLL motif on TRIM24 interacts with retinoic acid receptor α (RARα) in a ligand-dependent manner to attenuate RARα-mediated gene transcription^17,18^. The C-terminus of TRIM24 contains a tandem Plant Homeodomain (PHD) and Bromodomain (Bromo), which function as a histone reader to recognise a combination of unmethylated H3K4 and acetylated H3K23 within a single histone tail^19^. This combinatorial readout of histone modifications and the binding of TRIM24 to oestrogen receptor activates a subset of oestrogen-dependent genes^19^. Besides its role in transcriptional regulation, TRIM24 may also regulate protein ubiquitination through an E3-ligase domain at its N-terminus. For example, TRIM24 can ubiquitinate p53 for degradation in both Drosophila and human breast cancer cells^20^. However, the physiological significance and molecular substrates of the E3-ligase of TRIM24 are still not fully understood.

This study demonstrates that through phosphorylation by PKB/Akt, insulin shuttles TRIM24 from the nucleus into the cytoplasm, where TRIM24 interacts with P-bodies to control hepatic peroxisome proliferator-activated receptor gamma (*Pparγ)* mRNA for regulation of hepatic lipogenesis.

## Results

### TRIM24 is an insulin-regulated phospho-protein in the liver

**Fig. 1:**
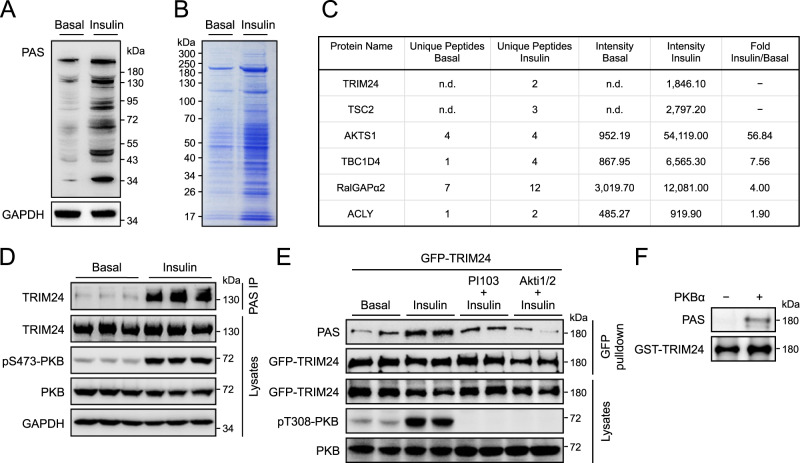
Identification of TRIM24 as a PKB substrate. **A** PAS-reactive phosphorylated proteins in mouse liver in response to insulin stimulation. PAS-reactive phosphorylated proteins were detected in mouse liver lysates with the PAS antibody using GAPDH as a loading control. **B** PAS-reactive phosphorylated proteins immunoprecipitated from mouse liver lysates. PAS-reactive phosphorylated proteins were immunoprecipitated from mouse liver lysates using the PAS antibody, separated via SDS-PAGE, and stained with Coomassie blue dye. Immunoprecipitated proteins were excised and subjected to identification via mass spectrometry. **C** PKB substrates identified via mass spectrometry in the liver. Numbers of unique peptides were listed, and signal intensities were quantified for each PKB substrate. n.d., not detectable. **D** PAS-reactive phosphorylation of TRIM24 in mouse liver in response to insulin. Phosphorylated proteins recognised by the PAS antibody were pulled down from lysates of mouse liver treated with or without insulin using a PAS antibody-conjugated resin. TRIM24 was detected in the immunoprecipitates via western blot using the specific antibodies. Quantitation results were shown in Supplementary Fig. 1A. **E** Effects of inhibitors of PI-3K and PKB on insulin-induced PAS-reactive phosphorylation of TRIM24. GFP-TRIM24 was expressed in HEK293 cells stimulated with or without insulin after pre-treatment with a PI-3K inhibitor PI-103, or a PKB inhibitor Akti1/2, or vehicle. After immunoprecipitated from cell lysates, phosphorylation of TRIM24 was determined using the PAS antibody. Quantitation results were shown in Supplementary Fig. 1B. **F** In vitro phosphorylation of GST-TRIM24 by recombinant PKBα. Phosphorylated GST-TRIM24 was detected using the PAS antibody. Source data are provided as a Source Data file.

To gain insights into how insulin regulates liver functions, we treated mice with insulin and detected phosphorylated proteins in liver lysates using a generic phospho-Akt substrate (PAS) antibody. As expected, insulin-stimulated PAS-reactive phosphorylation of a number of proteins in the liver (Fig. 1A). We immunoprecipitated these PAS-reactive phospho-proteins using the immobilised PAS antibody and, by mass spectrometry, identified over 400 proteins enriched in the insulin-stimulated sample (Fig. 1B, Supplementary Data 1), including several known PKB/Akt substrates such as TSC2, PRAS40/AKTS1, AS160/TBC1D4, RalGAPα2, and ACLY (Fig. 1C, Supplementary Data 1). TRIM proteins have been found as regulators of the PI-3K − PKB pathway^21^. However, it is not clear whether and how the insulin−PI-3K–PKB pathway in turn might regulate TRIM proteins via their direct phosphorylation although some TRIM proteins such as TRIM24 have been identified as potential PAS-reactive proteins in previous proteomics studies^22,23^. Of relevance, TRIM24 was also identified as a potential phosphorylated protein in reponse to insulin in our proteomics study as its abundance was increased in the PAS immunoprecipitates upon insulin stimulation (Fig. 1C, Supplementary Data 1). The presence of TRIM24 in the PAS immunoprecipitates was confirmed via immunoblotting using a TRIM24-specific antibody (Fig. 1D, Supplementary Fig. 1A). Using a GFP-TRIM24 fusion protein expressed in HEK293 cells, we found its PAS-reactive phosphorylation increased upon stimulation with insulin (Fig. 1E, Supplementary Fig. 1B). This insulin-stimulated PAS-reactive phosphorylation of TRIM24 was blunted when cells were pre-treated with either the PI-3K inhibitor PI-103 or PKB/Akt inhibitor Akti1/2 (Fig. 1E, Supplementary Fig. 1B). Moreover, in an in vitro assay the recombinant PKB/Akt phosphorylated TRIM24 could be detected with the PAS antibody (Fig. 1F). These data demonstrate that TRIM24 is a PKB/Akt substrate, and that insulin stimulates its phosphorylation.

### Insulin translocates TRIM24 from the nucleus into the cytosol through its Ser^1043^ phosphorylation

**Fig. 2:**
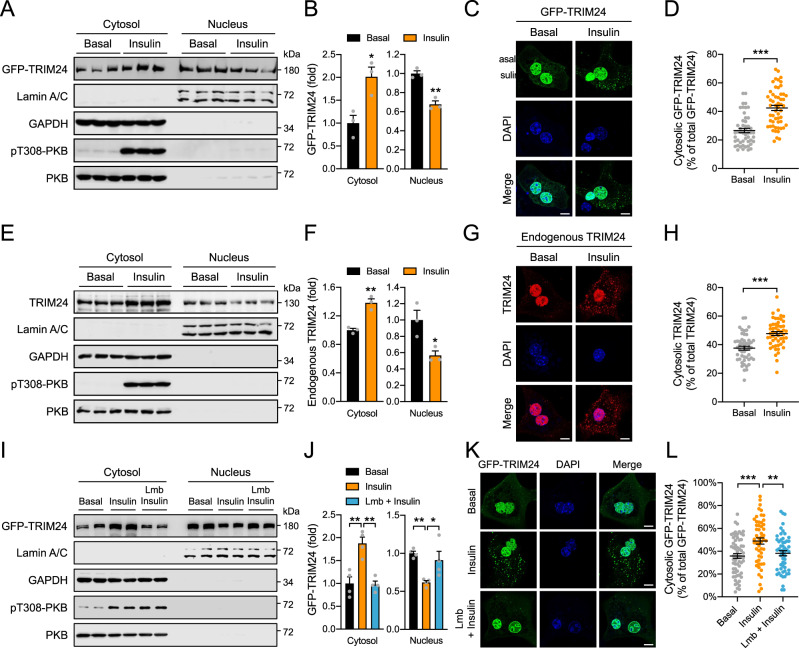
Insulin-induced cytosolic localisation of TRIM24. **A**, **B** Subcellular distribution of GFP-TRIM24 in HEK293 cells in response to insulin. GAPDH was used as a cytosolic marker, and Lamin A/C as a nuclear marker. **A** immunoblots. **B** quantitative results. *n* = 3. *p* = 0.020 (Cytosol) and 0.0023 (Nucleus). **C, D** Subcellular localisation of GFP-TRIM24 in response to insulin. Mouse primary hepatocytes were transfected with GFP-TRIM24-expressing plasmids and subsequently stimulated with or without insulin. **C** Representative images. **D** Quantitative results. *n* = 50 (Basal) and 54 (insulin). *p* = 1.10e−9. **E**, **F** Subcellular distribution of endogenous TRIM24 in mouse primary hepatocytes in response to insulin. Primary hepatocytes were treated with or without insulin and subjected to cellular fractionation. GAPDH was detected as a cytosolic marker, and Lamin A/C as a nuclear marker. **E** immunoblots. **F** quantitative results. *n* = 3. *p* = 0.0030 (Cytosol) and 0.030 (Nucleus). **G**, **H** Subcellular localisation of endogenous TRIM24 in mouse primary hepatocytes in response to insulin. **G** representative images. **H** quantitative results. *n* = 50 (Basal) and 52 (Insulin). *p* = 6.12e−7. **I**, **J** Subcellular distribution of GFP-TRIM24 in HEK293 cells in response to insulin and exportin inhibitor Leptomycin B (Lmb). GAPDH was used as a cytosolic marker, and Lamin A/C as a nuclear marker. I, representative immunoblots. **J** Quantitative results. *n* = 4. *p* = 0.0017 (Cytosol, Insulin vs Basal), 0.0014 (Cytosol, Insulin vs Lmb+Insulin), 0.0099 (Nucleus, Insulin vs Basal) and 0.039 (Nucleus, Insulin vs Lmb + Insulin). **K**, **L** Subcellular localisation of GFP-TRIM24 in mouse primary hepatocytes in response to insulin and Lmb. **K** representative images. **L** quantitative results. *n* = 63 (Basal), 53 (Insulin) and 54 (Lmb + Insulin). *p* = 0.0003 (Insulin vs Basal) and 0.0055 (Insulin vs Lmb+Insulin). Scale bars in **C**, **G**, **K** indicate 10 μm in length. Data are given as the mean ± SEM. Statistical analyses were carried out via two-sided *t*-test for **B**, **D**, **F**, **H**, and via one-way ANOVA for **J**, **L**. * indicates *p* < 0.05, ** indicates *p* < 0.01, and *** indicates *p* < 0.001. Source data are provided as a Source Data file.

TRIM24 is generally considered a nuclear protein that has two predicted monopartite nucleus localisation signals (NLS), PIDKRKCERL (NLS1, position 903-912aa, residue numbering according to mouse TRIM24) and VQPRKKRLKSIEE (NLS2, position 1034-1046aa) (www.NLS-mapper.lab.keio.ac.jp). Of note, a substantial amount of GFP-TRIM24 was detected in the cytosolic fraction of cells when expressed in HEK293 cells (Fig. 2A, B). Moreover, insulin stimulation decreased the nuclear abundance of GFP-TRIM24 and increased its levels in the cytosol (Fig. 2A, B). Fluorescence imaging also showed that under the basal conditions, GFP-TRIM24 was mainly found in the nucleus, and that in response to insulin stimulation, a portion of GFP-TRIM24 translocated from the nucleus into the cytosol forming puncta dispersed in the cytosol (Fig. 2C, D). Furthermore, endogenous TRIM24 in the cytosol of primary hepatocytes was also significantly increased and formed cytosolic puncta structures when cells were stimulated with insulin (Fig. 2E–H). Notably, treatment with a nuclear export inhibitor leptomycin B (Lmb) blocked the insulin-induced increase of cytosolic GFP-TRIM24 and simultaneously prevented the decrease of nuclear GFP-TRIM24 upon insulin stimulation (Fig. 2I–L). Therefore, the elevation of cytosolic GFP-TRIM24 was most likely due to an increase of its export from the nucleus rather than a decrease of its import from the cytosol upon insulin stimulation. In agreement, we found that GFP-TRIM24 interacted with Flag-tagged exportin-1 (XPO1) when they were co-expressed in cells (Supplementary Fig. 2A). Co-expression of Flag-XPO1 increased the level of cytosolic GFP-TRIM24 while decreased the nuclear level of GFP-TRIM24 (Supplementary Fig. 2B).

**Fig. 3:**
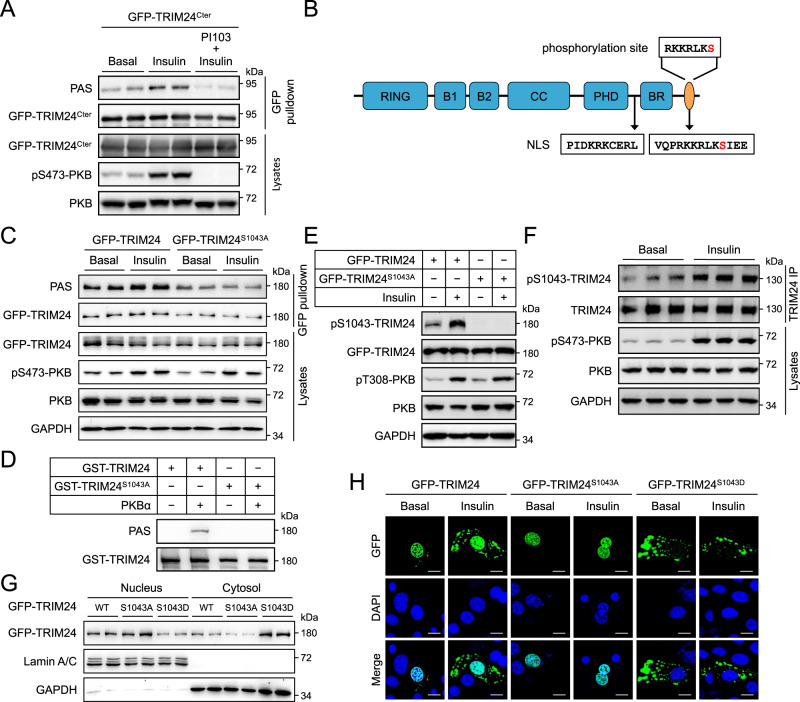
Phosphorylation-dependent cytosolic localisation of TRIM24. **A** PAS-reactive phosphorylation of a C-terminal fragment TRIM24^Cter^. GFP-TRIM24^Cter^ (spanning from G601-end of TRIM24) was expressed in HEK293 cells stimulated with or without insulin after pre-treatment with PI-103. PAS-reactive phosphorylation was detected on immunoprecipitated GFP-TRIM24^Cter^ using the PAS antibody. Total and phosphorylated PKB were determined in cell lysates. **B** Diagrammatic illustration of the Ser^1043^ site and two NLSs on TRIM24. Ser^1043^ is highlighted in red. **C** Effects of Ser^1043^ mutation on insulin-induced PAS-reactive phosphorylation of TRIM24. WT GFP-TRIM24 and mutant GFP-TRIM24^S1043A^ were expressed in HEK293 cells that were stimulated with or without insulin. After immunoprecipitated from cell lysates, phosphorylation of TRIM24 was determined using the PAS antibody. **D** In vitro phosphorylation of WT GST-TRIM24 and mutant GST-TRIM24^S1043A^ by a recombinant PKBα. Phosphorylated GST-TRIM24 was detected using the PAS antibody. **E** Ser^1043^ phosphorylation on GFP-TRIM24 in response to insulin. WT GFP-TRIM24 and mutant GFP-TRIM24^S1043A^ were expressed in HEK293 cells that were stimulated with or without insulin. Ser^1043^ phosphorylation on GFP-TRIM24 was determined using the site-specific phospho-antibody. **F** Ser^1043^ phosphorylation on endogenous TRIM24 in response to insulin. Mouse primary hepatocytes were stimulated with or without insulin. Endogenous TRIM24 was immunoprecipitated from cell lysates and Ser^1043^ phosphorylation on it was determined using the site-specific phospho-antibody. Quantitation results were shown in Supplementary Fig. 1E. **G** Subcellular distribution of GFP-TRIM24 WT, S1043A and S1043D mutants. GFP-TRIM24 proteins were expressed HEK293 cells, and their subcellular distribution was determined in the nuclear and cytosolic fractions via immunoblotting. GAPDH was used as a cytosolic marker while Lamin A/C was detected as a nuclear marker. **H** Subcellular localisation of GFP-TRIM24 WT and mutant proteins in response to insulin. GFP-TRIM24 WT, S1043A and S1043D mutants were expressed mouse primary hepatocytes that were stimulated with or without insulin. After fixation, cells were stained with DAPI and photographed using a confocal microscope. Scale bars indicate 10 μm in length. Source data are provided as a Source Data file.

These data led us to hypothesise that phosphorylation of TRIM24 may govern its cytosolic translocation in response to insulin. We found that the insulin-induced PAS-reactive phosphorylation site(s) resides on the C-terminal region of TRIM24 (Fig. 3A). Among the possible phosphorylation sites (www.phosphosite.org), Ser^1043^ (numbering according to mouse sequence) and its surrounding sequence (RKKRLK**s**, Ser^1043^ is shown in lower case bold) conform to the binding motif (RXRXXpS/T) of the PAS antibody (Fig. 3B). This sequence is conserved in mammals (Supplementary Fig. 1C). Substitution of Ser^1043^ with a non-phosphorylatable alanine blocked insulin/PKB-mediated PAS-reactive phosphorylation of TRIM24 *in cellules* as well as in vitro (Fig. 3C, D). We then raised a site-specific antibody recognising phosphorylated Ser^1043^ (pSer^1043^) on TRIM24, whose specificity was validated via a dot blot using a pSer^1043^-TRIM24 peptide and a corresponding non-phosphopeptide (Supplementary Fig. 1D). Indeed, insulin markedly increased Ser^1043^ phosphorylation on GFP-TRIM24, which was prevented when this site was mutated to alanine (Fig. 3E). Accordingly, insulin significantly increased Ser^1043^ phosphorylation on endogenous TRIM24 in the liver (Fig. 3F, Supplementary Fig. 1E). When Ser^1043^ was mutated to a phospho-mimic aspartate, the GFP-TRIM24^S1043D^ mutant protein was found predominantly in the cytosol (Fig. 3G, H). In contrast, the GFP-TRIM24^S1043A^ mutant protein was mainly present in the nucleus, and insulin could no longer translocate this mutant protein from the nucleus into the cytosol (Fig. 3G, H). Notably, the GFP-TRIM24^S1043D^ mutant protein formed cytosolic foci, regardless of insulin stimulation, similar to the insulin-induced cytosolic GFP-TRIM24 (Fig. 3H). Together, these data show that insulin promotes the translocation of TRIM24 from the nucleus into the cytosol through its Ser^1043^ phosphorylation.

Interestingly, Ser^1043^ is located within the predicted NLS2 of TRIM24, which could indeed target a GFP-GST fusion protein to the nucleus (Supplementary Fig. 2E). Similar as the GFP-TRIM24^S1043D^ mutant protein, the GFP-GST-NLS2^S1043D^ protein was also predominantly expressed in the cytosol but as a diffused form (Supplementary Fig. 2E). We then mutated the basic residues on NLS2 to alanine to identify critical residues for NLS2 to direct nucleus targeting. Mutation of Arg^1037^ and Lys^1038^ did not affect the nuclear localisation of GFP-GST-NLS2 while mutation of Lys^1039^ or Arg^1040^ or Lys^1042^ resulted in its cytosolic localisation (Supplementary Fig. 2E). Insulin stimulated the PAS-reactive phosphorylation on wild-type (WT) GFP-GST-NLS2, which was blocked by the K1039A and R1040A mutations on NLS2 (Supplementary Fig. 2F). In contrast, the PAS-reactive phosphorylation became constitutively elevated on the GFP-GST-NLS2^K1042A^ mutant as compared to the WT GFP-GST-NLS2 (Supplementary Fig. 2F). The GFP-TRIM24^K1039A^, GFP-TRIM24^R1040A^ and GFP-TRIM24^K1042A^ formed cytosolic puncta in a manner similar to GFP-TRIM24^S1043D^ (Supplementary Fig. 2G), suggesting that the cytosolic presence of GFP-TRIM24 rather than Ser^1043^ phosphorylation per se led to cytosolic puncta formation. Since Ser^1043^ phosphorylation was required for insulin-stimulated export of GFP-TRIM24 (Fig. 3G), we wondered whether the NLS2 might bind to XPO1 as the full-length protein did (Supplementary Fig. 2A). Indeed, GFP-GST-NLS2 was found in the immunoprecipitates of Flag-XPO1 when the two proteins were co-expressed in cells (Supplementary Fig. 2C). Moreover, Flag-XPO1 was also detected in the immunoprecipitates of GFP-GST-NLS2 in a reciprocal immunoprecipitation experiment (Supplementary Fig. 2D).

### TRIM24 interacts with critical components of P-bodies in the cytosol

**Fig. 4:**
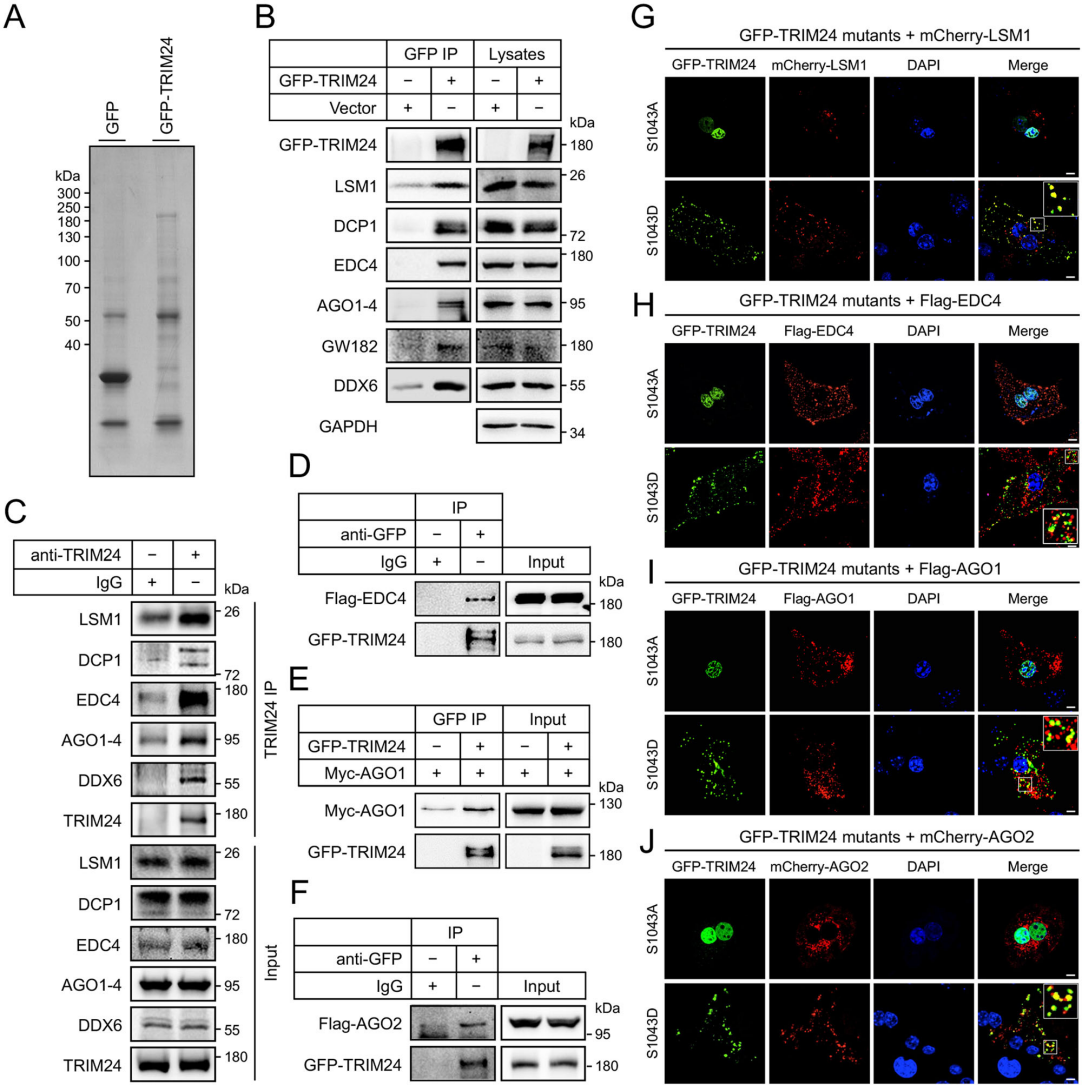
Interaction of cytosolic TRIM24 with P-bodies. **A** Immunoprecipitation of GFP-TRIM24. GFP-TRIM24 and free GFP were expressed in HEK293 cells, and immunoprecipitated from cell lysates using the GFP-Trap®-agarose. Immunoprecipitates were separated via SDS-PAGE, stained with Coomassie blue dye, and subjected to identification via mass spectrometry. **B** Co-immunoprecipitation of P-body components with GFP-TRIM24. GFP-TRIM24 was expressed in HEK293 cells, and immunoprecipitated from cell lysates using the GFP antibody. Endogenous P-body components were detected in the GFP-TRIM24 immunoprecipitates and cell lysates via immunoblotting using the specific antibodies. **C** Co-immunoprecipitation of P-body components with endogenous TRIM24. TRIM24 was immunoprecipitated from lysates of mouse primary hepatocytes using the TRIM24 antibody. Endogenous P-body components were detected in the TRIM24 immunoprecipitates and cell lysates via immunoblotting using the specific antibodies. **D** Interaction of Flag-EDC4 and GFP-TRIM24. Flag-EDC4 and GFP-TRIM24 were co-expressed in HEK293 cells, and immunoprecipitation was performed using the GFP antibody or an IgG. Flag-EDC4 and GFP-TRIM24 were detected in the immunoprecipitates and cell lysates via immunoblotting using the specific antibodies. **E** Interaction of Myc-AGO1 and GFP-TRIM24. Myc-AGO1 was co-expressed with or without GFP-TRIM24 in HEK293 cells, and immunoprecipitation was performed using the GFP antibody. Myc-AGO1 and GFP-TRIM24 were detected in the immunoprecipitates and cell lysates via immunoblotting using the specific antibodies. **F** Interaction of Flag-AGO2 and GFP-TRIM24. Flag-AGO2 and GFP-TRIM24 were co-expressed in HEK293 cells, and immunoprecipitation was performed using the GFP antibody or an IgG. Flag-AGO2 and GFP-TRIM24 were detected in the immunoprecipitates and cell lysates via immunoblotting using the specific antibodies. **G**–**J** Co-localisation of GFP-TRIM24 proteins with P-body components. GFP-TRIM24^S1042A^ and GFP-TRIM24^S1042D^ were co-expressed with mCherry-LSM1 (**G**), Flag-EDC4 (**H**), Flag-AGO1 (**I**) and mCherry-AGO2 (**J**) in mouse primary hepatocytes. After fixation, cells were stained with DAPI and the Flag antibody in case of Flag-EDC4 and Flag-AGO1. Images were taken using a confocal microscope. The regions highlighted were enlarged in the insets. Scale bars indicate 10 μm in length. Source data are provided as a Source Data file.

To understand the potential roles of TRIM24 in the cytosol, we first examined its co-localisation with organelles in primary hepatocytes. The foci of GFP-TRIM24^S1043D^ mutant protein did not colocalise with the mitochondria, endoplasmic reticulum, Golgi apparatus, endosomes and lysosomes (Supplementary Fig. 3A–F). We then sought to identify protein interactors of TRIM24 via a proteomic approach, which we reasoned might help reveal its cytosolic function. To this end, we expressed the GFP-TRIM24 fusion protein in HEK293 cells and immunoprecipitated it from the cell lysates using the GFP antibody (Fig. 4A). Proteins in the immunoprecipitates were identified by mass spectrometry, among which XPO1 was found (63 peptides identified). We found many P-body components in the TRIM24 immunoprecipitates, including LSM1, EDC4, AGOs, and GW182 (Supplementary Data 2). We verified via western blots the presence of these proteins and also two other markers of P-bodies, DDX6 and DCP1, in the GFP-TRIM24 immunoprecipitates (Fig. 4B). More importantly, endogenous LSM1, EDC4, AGOs, DCP1 and DDX6 were detected in the immunoprecipitates of endogenous TRIM24 from liver lysates (Fig. 4C). We then focused on EDC4, AGO1, and AGO2, fused these proteins with a Flag or Myc tag, and co-expressed with the GFP-TRIM24 in HEK293 cells. Co-immunoprecipitation of these fusion proteins again demonstrated the interaction between GFP-TRIM24 and these P-body components (Fig. 4D–F). Furthermore, cytosolic GFP-TRIM24^S1043D^, but not nuclear GFP-TRIM24^S1043A^, was colocalised with co-expressed mCherry-LSM1, and was detected in part in Flag-EDC4, Flag-AGO1, and mCherry-AGO2 foci (Fig. 4G–J). The foci formed by GFP-LSM1, Flag-EDC4 and mCherry-AGO2 exhibited partial co-localisation with each other (Supplementary Fig. 4A–C), suggesting that P-bodies might have sub-populations. These data suggested that P-bodies might be heterogeneous and cytosolic TRIM24 might associate with some sub-populations of P-bodies.

Stress granules are another type of non-membraneous organelles involving in mRNA storage and/or decay^8^. In contrast to P-bodies, cytosolic GFP-TRIM24^S1043D^ and nuclear GFP-TRIM24^S1043A^ did not colocalise with mCherry-G3BP1, a marker for stress granules, suggesting that TRIM24 might not regulate stress granules (Supplementary Fig. 5).

### TRIM24 regulates EDC4 and AGO2 through poly-ubiquitination

**Fig. 5:**
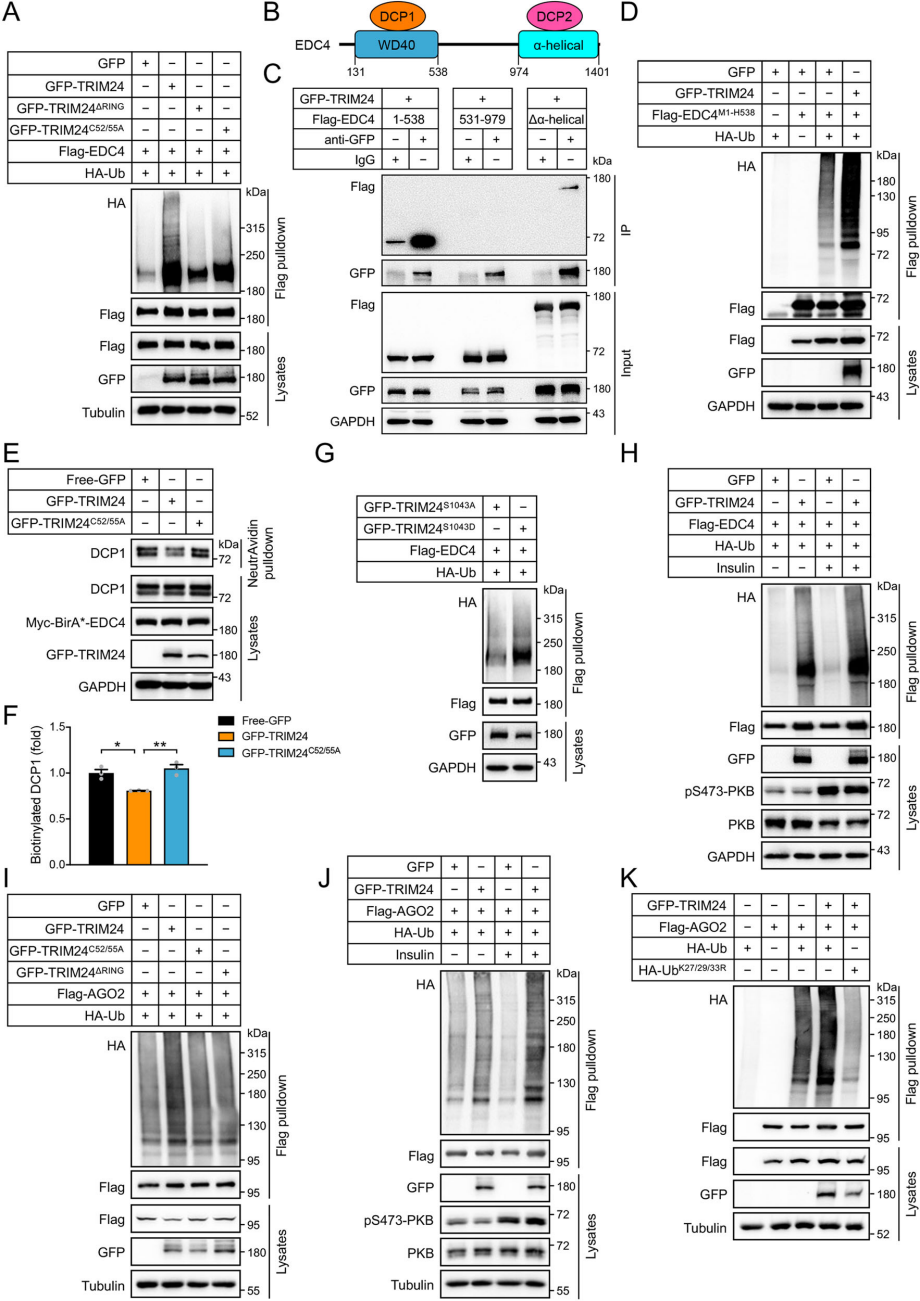
Interaction and ubiquitination of EDC4 and AGO2 by TRIM24. Interaction and ubiquitination assays were performed in HEK293 cells expressing tagged proteins of interest. After immunoprecipitation, poly-ubiquitination of proteins of interest by HA-Ub was detected via immunoblotting using the HA antibody. **A** Ubiquitination of Flag-EDC4 co-expressed with GFP, or GFP-TRIM24, GFP-TRIM24^ΔRING^, or GFP-TRIM24^C52/55A^ in the presence of HA-Ub. **B** Diagrammatic illustration of domain compositions of EDC4 and its interaction with DCP1 and DCP2. DCP1 interacts with the WD40 domain on EDC4, and DCP2 binds to the α-helical region of EDC4. **C** Mapping of interaction domains of EDC4 with TRIM24. GFP-TRIM24 was immunoprecipitated using the GFP antibody, and Flag-tagged domains of EDC4 were detected in the immunoprecipitates. **D** Ubiquitination of Flag-EDC4^M1-H538^ co-expressed with GFP, or GFP-TRIM24 in the presence of HA-Ub. **E**, **F** BioID assay of interaction between DCP1 and EDC4. Free-GFP, or GFP-TRIM24 or GFP-TRIM24^C52/55A^ was expressed in Myc-BirA*-EDC4 expressing HEK293 cells. Biotinylated proteins were pulled down using the NeutrAvidin beads, DCP1 and Myc-BirA*-EDC4 were detected in the precipitates via immunoblotting using the DCP1 and Myc antibodies, respectively. **E** Representative blots. **F** Quantitation of biotinylated DCP1 in the BioID assay. *n* = 3. *p* = 0.013 (GFP-TRIM24 vs Free GFP) and 0.0043 (GFP-TRIM24 vs GFP-TRIM24^C52/55A^). **G** Ubiquitination of Flag-EDC4 co-expressed with GFP-TRIM24^S1043A^ or GFP-TRIM24^S1043D^ in the presence of HA-Ub. **H** Ubiquitination of EDC4 by GFP-TRIM24 in response to insulin. **I** Ubiquitination of Flag-AGO2 co-expressed with GFP, GFP-TRIM24, GFP-TRIM24^ΔRING^, or GFP-TRIM24^C52/55A^ the presence of HA-Ub. **J** Ubiquitination of AGO2 by GFP-TRIM24 in response to insulin. **K** Chain type of ubiquitination of AGO2 by TRIM24. Flag-AGO2 was co-expressed with GFP-TRIM24 in HEK293 cells in the presence of HA-Ub or HA-Ub^K27/29/33R^. Data are given as the mean ± SEM. Statistical analyses were carried out via one-way ANOVA. * indicates *p* < 0.05, and ** indicates *p* < 0.01. Source data are provided as a Source Data file.

The mRNA decapping complex consists of EDC4, a decapping enzyme DCP2 and its coactivator DCP1. EDC4 functions as a scaffold that simultaneously interacts with DCP1 and DCP2 facilitating the association of these two proteins^24^. TRIM24 is a multi-functional protein possessing a RING-type E3-ligase domain, which prompted us to investigate whether it might ubiquitinate EDC4. Indeed, co-expression of TRIM24 with EDC4 in HEK293 cells increased ubiquitination of EDC4 protein (Fig. 5A). In contrast, a RING-domain deletion TRIM24^ΔRING^ mutant or a ligase-dead TRIM24^C52/55A^ mutant protein (Cys52 and Cys55, critical residues in the RING E3-ligase domain of TRIM24 were mutated to alanine) displayed impairment in their abilities to ubiquitinate EDC4 (Fig. 5A). Mutation analyses revealed that TRIM24 ubiquitinated EDC4 through multiple chain linkages of K6, K27, K29 and K33 (Supplementary Fig. 6A, B). We then mapped the TRIM24-interacting region on EDC4 through fragmentation analysis and found that an EDC4^M1-H538^ fragment containing a WD40 domain interacted with TRIM24 (Fig. 5B, C). Moreover, co-expression of TRIM24 enhanced ubiquitination of the EDC4^M1-H538^ fragment (Fig. 5D). This WD40 domain is the binding site of DCP1 on the scaffold EDC4 in the decapping complex^24^. We, therefore, hypothesised that TRIM24-mediated ubiquitination of EDC4 might inhibit the assembly of DCP1 on EDC4. To test this hypothesis, we employed a proximity-dependent biotin identification (BioID) assay by expressing Myc-BirA*-EDC4 in HEK293 cells together with GFP-TRIM24, or GFP-TRIM24^C52/55A^, or GFP. DCP1 was as expected biotinylated by Myc-BirA*-EDC4, and its biotinylation was decreased in the presence of GFP-TRIM24 as compared to co-expression with GFP (Fig. 5E, F). In contrast, co-expression with GFP-TRIM24^C52/55A^ did not decrease the biotinylation of DCP1 by Myc-BirA*-EDC4 (Fig. 5E, F). These data indicate that ubiquitination of EDC4 by TRIM24 may inhibit assembly of the decapping complex through dissociating DCP1 from the EDC4 scaffold. We then examined whether insulin-mediated TRIM24 translocation might increase EDC4 ubiquitination. Co-expression of phospho-mimic GFP-TRIM24^S1043D^ mutant elevated ubiquitination of Flag-EDC4 as compared to the non-phosphorylatable GFP-TRIM24^S1043A^ mutant (Fig. 5G). Moreover, insulin increased ubiquitination of Flag-EDC4 when co-expressed with GFP-TRIM24 (Fig. 5H).

Similarly, we found that TRIM24 also ubiquitinated AGO2 and that RING-domain deletion or C52/55 A mutation of TRIM24 inhibited ubiquitination of AGO2 (Fig. 5I). Insulin stimulation also enhanced ubiquitination of Flag-AGO2 when co-expressed with GFP-TRIM24 (Fig. 5J). TRIM24-mediated ubiquitination of AGO2 involved chain linkages of K27, K29 and K33, and simultaneous mutation of these three lysine residues to arginine diminished ubiquitination of AGO2 by TRIM24 (Fig. 5K, Supplementary Fig. 7A, B). AGO2 is a multi-domain containing protein (Supplementary Fig. 7C). An AGO2^M1-D480^ fragment containing ArgoN, ArgoL and PAZ domains strongly interacted with TRIM24, while an AGO2^S478-X860^ fragment harbouring a Piwi domain also bound to TRIM24 yet to a lesser extent (Supplementary Fig. 7D).

### Inhibition of TRIM24 E3-ligase activity down-regulates hepatic expression of lipogenic genes and attenuates diet-induced hepatosteatosis

**Fig. 6:**
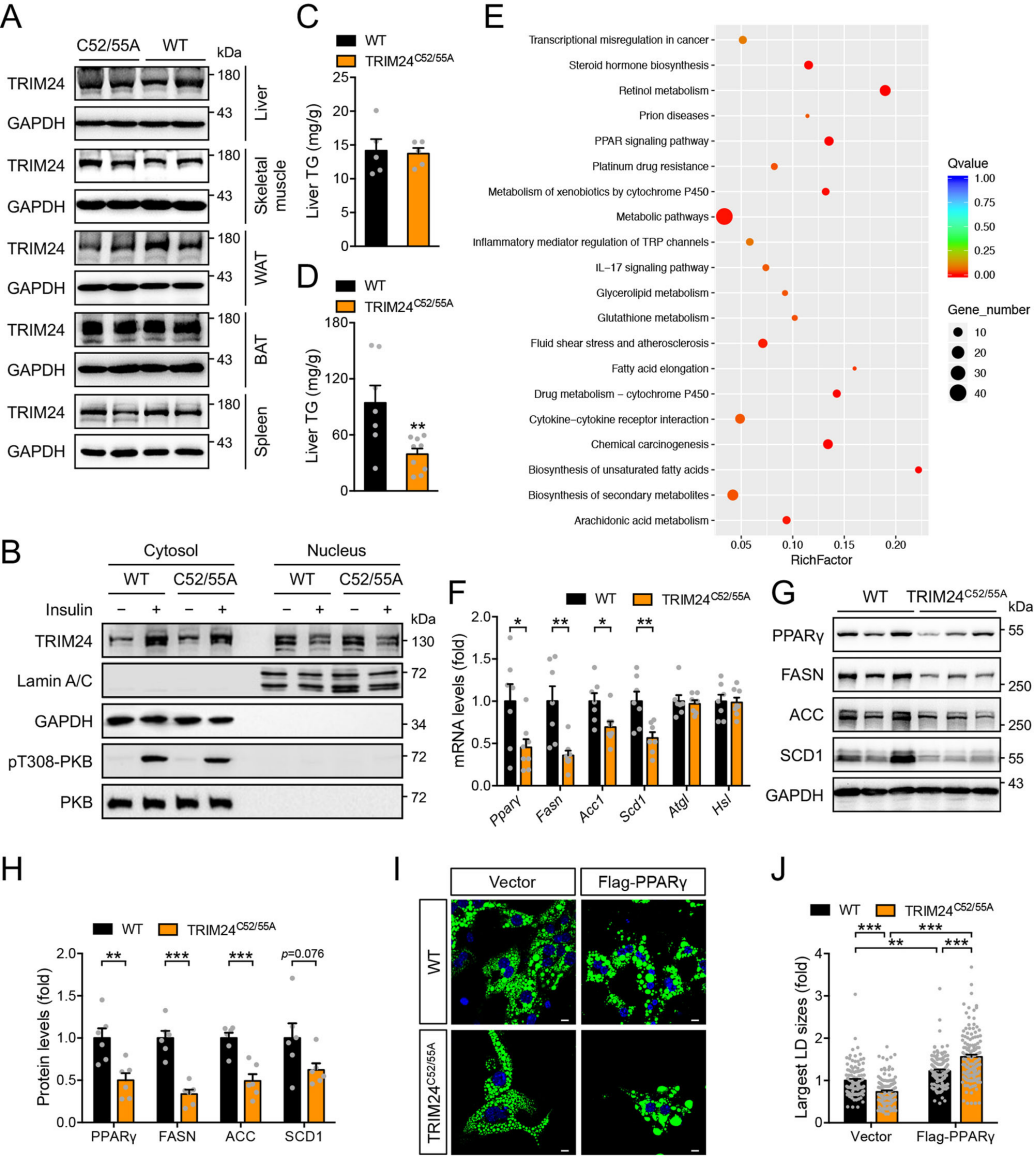
Hepatic lipid metabolism of the TRIM24^C52/55A^ mice. **A** Expression of TRIM24 protein in various tissues of the 2.5-month-old female TRIM24^C52/55A^ and WT mice. **B** Subcellular distribution of endogenous TRIM24 in TRIM24^C52/55A^ and WT hepatocytes in response to insulin. After cellular fractionation, subcellular distribution of TRIM24 was measured in the nuclear and cytosolic fractions via immunoblotting. GAPDH was detected as a cytosolic marker, and Lamin A/C as a nuclear marker. Representative blots were shown here. Quantitation results were shown in Supplementary Fig. 8C. **C** Liver TG in 5-month-old male TRIM24^C52/55A^ and WT mice fed the CD. *n* = 5. **D** Liver TG in 5-month-old male TRIM24^C52/55A^ and WT mice fed the HFD. *n* = 7 (WT) and 9 (TRIM24^C52/55A^). *p* = 0.0075. **E** KEGG pathway analysis of differentially-expressed genes in the liver of HFD-fed TRIM24^C52/55A^ and WT mice. **F** Expression of lipid metabolic genes in the liver of 5-month-old male TRIM24^C52/55A^ and WT mice on HFD. *n* = 7 (WT) and 8 (TRIM24^C52/55A^). In case of *Scd1* and *Atgl*, *n* = 7 for TRIM24^C52/55A^. *p* = 0.025 (*Pparγ*), 0.0027 (*Fasn*), 0.018 (*Acc1*), 0.0062 (*Scd1*), 0.73 (*Atgl*) and 0.90 (*Hsl*). **G**, **H**. Protein levels of PPARγ, FASN, ACC, and SCD1 in the liver of 5-month-old male TRIM24^C52/55A^ and WT mice on HFD. G, representative blots. **H** Quantitation data. *n* = 6. *p* = 0.0057 (PPARγ), 0.000054 (FASN), 0.00056 (ACC) and 0.076 (SCD1). **I**, **J** LDs in TRIM24^C52/55A^ and WT primary hepatocytes transfected with empty vector or Flag-PPARγ. LDs in hepatocytes were stained with Bodipy. **I** Representative images. **J** Quantitation of the largest LDs in primary hepatocytes. Scale bars indicate 10 μm in length. *n* = 110 (WT/Vector), 120 (WT/Flag-PPARγ), 114 (TRIM24^C52/55A^/Vector) and 127 (TRIM24^C52/55A^/Flag-PPARγ). *p* = 0.0010 (WT/Vector vs WT/Flag-PPARγ), and *p* < 0.0001 for the rest. Data are given as the mean ± SEM. Statistical analyses were carried out via two-sided *t*-test for **C**, **D**, **F**, **H**, and via two-way ANOVA for **J**. * indicates *p* < 0.05, ** indicates *p* < 0.01, and *** indicates *p* < 0.001. Source data are provided as a Source Data file.

To study the in vivo role of TRIM24 in regulating the P-bodies, we generated a TRIM24^C52/55A^ knockin mouse model in which Cys52 and Cys55 on the endogenous TRIM24 were substituted by alanine (Supplementary Fig. 8A, B). The knockin mutations did not affect the expression of the mutant TRIM24 protein in mice (Fig. 6A). Insulin increased cytosolic abundance of TRIM24 and simultaneously decreased its nuclear level in primary hepatocytes from the WT control mice (Fig. 6B, Supplementary Fig. 8C, D). Similar insulin-induced translocation was observed for the mutant TRIM24 in primary hepatocytes from the TRIM24^C52/55A^ knockin mice (Fig. 6B, Supplementary Fig. 8C, D). These TRIM24^C52/55A^ knockin mice had normal serum triglyceride (TG) on both chow diet and high-fat diet (HFD) (Supplementary Fig. 8E, F). Although the TRIM24^C52/55A^ knockin mutation did not affect hepatic TG content when mice were fed with the chow diet (Fig. 6C), it significantly decreased HFD-induced hepatic TG accumulation and alleviated HFD-elicited hepatosteatosis in mice (Fig. 6D).

We then carried out transcriptomic analysis via deep sequencing of RNA (RNA-Seq) in the liver of HFD-fed mice (Supplementary Data 3) and found that PPAR signalling and fatty acid metabolism pathways were downregulated in the TRIM24^C52/55A^ liver (Fig. 6E, Supplementary Fig. 8G). We further confirmed the decreases of *Pparγ*, *Fasn*, *Acc1* and *Scd1* in the TRIM24^C52/55A^ liver via Q-PCR (Fig. 6F), parallelled with the decrease of their corresponding proteins (Fig. 6G, H). In contrast, mRNA levels of two lipases ATGL and HSL, and four other lipogenic transcription factors, namely SREBP1, ChREBP1, LXRα and USF1, exhibited no alterations in RNA-Seq, which was further confirmed via Q-PCR (Fig. 6F, Supplementary Fig. 8H). At the protein level, both full-length SREBP1 (flSREBP1) and N-terminal SREBP1 (nSREBP1, transcriptionally active) displayed no difference between the TRIM24^C52/55A^ and WT mice (Supplementary Fig. 9A). The key regulators of fatty acid oxidation, PPARα and CPT1, also remained normal in the TRIM24^C52/55A^ liver (Supplementary Fig. 9A). PPARγ is a critical regulator of hepatic lipid metabolism, whose deficiency in the liver attenuates HFD-induced hepatosteatosis^25^. We then examined the importance of PPARγ in the control of lipid storage in primary hepatocytes from the TRIM24^C52/55A^ knockin mice. Interestingly, the sizes of lipid droplets (LDs) in which TGs are stored were smaller in primary TRIM24^C52/55A^ hepatocytes than in WT hepatocytes (Fig. 6I, J). Significantly, overexpression of PPARγ restored the sizes of LDs in primary TRIM24^C52/55A^ knockin hepatocytes to a level even higher than those in WT cells (Fig. 6I, J). These data demonstrate that the E3-ligase activity of TRIM24 regulates the expression of critical genes in PPARγ signalling and fatty acid biosynthetic pathways, and its deficiency alleviates of HFD-induced hepatosteatosis through downregulation of the PPARγ pathway.

### TRIM24 regulates mRNA stability of *Pparγ* via the P-bodies

**Fig. 7:**
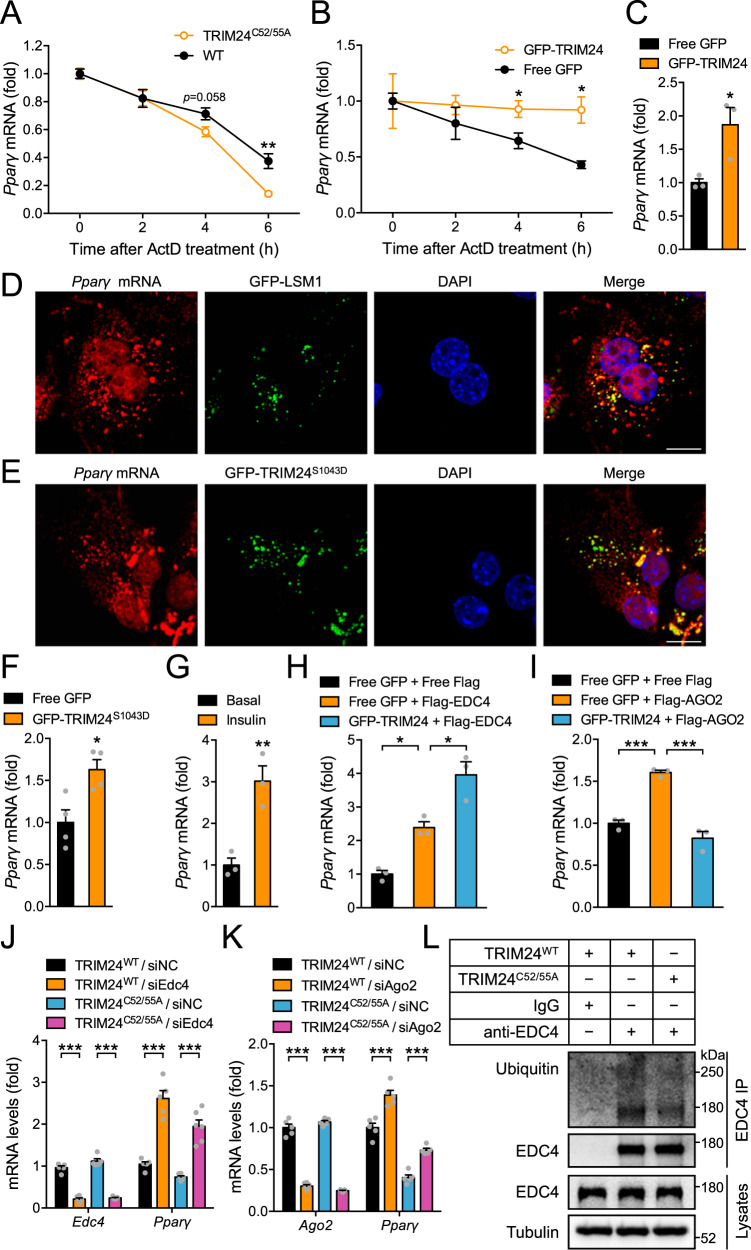
Regulation of *Pparγ* stability by TRIM24 via P-bodies. **A** Stability of *Pparγ* mRNA in TRIM24^C52/55A^ and WT hepatocytes upon treatment with actinomycin-D (ActD). *n* = 4. *p* = 0.0051 (TRIM24^C52/55A^/6 h vs WT/6 h). **B** Stability of *Pparγ* mRNA in mouse primary hepatocytes expressing GFP-TRIM24 or free GFP upon ActD treatment. *n* = 3. *p* = 0.0496 (GFP-TRIM24/4 h vs GFP/4 h) and 0.016 (GFP-TRIM24/6 h vs GFP/6 h). **C** *Pparγ* mRNA levels in mouse primary hepatocytes expressing GFP-TRIM24 or free GFP. *n* = 3. *p* = 0.030. D-E. *Pparγ* mRNA in situ hybridisation in mouse primary hepatocytes expressing GFP-LSM1 (**D**) or GFP-TRIM24^S1043D^ (**E**). Scale bars indicate 10 μm in length. **F** *Pparγ* mRNA in RNA immunoprecipitates (RIP) from mouse primary hepatocytes expressing GFP-TRIM24^S1043D^ or GFP through the GFP antibody. *n* = 4. *p* = 0.017. **G** *Pparγ* mRNA in RIP from insulin-treated mouse primary hepatocytes using the TRIM24 antibody. *n* = 3. *p* = 0.0076. **H** *Pparγ* mRNA in RIP from HEK293 cells co-expressing Flag-EDC4 and GFP-TRIM24 or GFP using the Flag antibody. n = 3. *p* = 0.020 (Flag-EDC4/GFP vs Flag/GFP) and 0.012 (Flag-EDC4/GFP-TRIM24 vs Flag-EDC4/GFP). **I** *Pparγ* mRNA in RIP in HEK293 cells co-expressing Flag-AGO2 and GFP-TRIM24 or GFP using the Flag antibody. *n* = 3. *p* = 0.0005 (Flag-AGO2/GFP vs Flag/GFP) and 0.0001 (Flag-AGO2/GFP-TRIM24 vs Flag-AGO2/GFP). **J**, **K** *Pparγ* mRNA levels in the TRIM24^C52/55A^ and WT hepatocytes upon downregulation of *Edc4* (**J**), or *Ago2* (**K**). **J** *n* = 5 (TRIM24^WT^/siNC, TRIM24^C52/55A^/siNC, TRIM24^C52/55A^/siEdc4) and 6 (TRIM24^WT^/siEdc4) for *Edc4*. *n* = 5 (TRIM24^WT^/siNC, TRIM24^WT^/siEdc4) and 6 (TRIM24^C52/55A^/siNC, TRIM24^C52/55A^/siEdc4) for *Pparγ*. *p* < 0.0001. K *n* = 5 (TRIM24^WT^/siNC, TRIM24^C52/55A^/siNC, TRIM24^C52/55A^/siAgo2) and 6 (TRIM24^WT^/siAgo2) for Ago2. *n* = 6 for *Pparγ*. *p* = 0.0004 (TRIM24^C52/55A^/siAgo2 vs TRIM24^C52/55A^/siNC), and *p* < 0.0001 for the rest. **L** Ubiquitination of endogenous EDC4 in TRIM24^C52/55A^ and WT hepatocytes. Data are given as the mean ± SEM. Statistical analyses: two-sided t-test for **A**–**C** and **F**, **G**, one-way ANOVA for **H**, **I**, and two-way ANOVA for **J**, **K**. * indicates *p* < 0.05, ** indicates *p* < 0.01, and *** indicates *p* < 0.001. Source data are provided as a Source Data file.

We next investigated how the inactivation of TRIM24 E3-ligase downregulated *Pparγ* at the mRNA level. The nuclear run-on experiment showed that transcription of *Pparγ* was increased in the liver of TRIM24^C52/55A^ knockin mice (Supplementary Fig. 8I). In contrast, stability of *Pparγ* mRNA was decreased in primary TRIM24^C52/55A^ hepatocytes compared to that in WT hepatocytes when cells were treated with actinomycin-D (ActD) (Fig. 7A). No TRIM24 binding site has been found at the *Pparγ* loci on the genome in a previous report^26^ although it is still possible that TRIM24 might indirectly affect *Pparγ* transcription via chromatin states. Nevertheless, our data suggest that the diminished *Pparγ* mRNA in the liver of TRIM24^C52/55A^ mice was most likely due to a decrease in its stability rather than transcription. In line with this possibility, overexpression of TRIM24 increased stability of *Pparγ* mRNA when transcription was blocked with ActD in cells, which led to an increase of *Pparγ* mRNA levels (Fig. 7B, C). In contrast, overexpression of TRIM24 did not alter stability of *Srebp1* mRNA in hepatocytes treated with ActD (Supplementary Fig. 10A). Since cytosolic TRIM24 was associated with the P-bodies, we wondered whether its effects on *Pparγ* mRNA depended on this membrane-less organelle. Interestingly, fluorescent in situ hybridisation showed that a portion of *Pparγ* mRNA was present in the cytosolic TRIM24 puncta as well as in LSM1-labelled P-bodies (Fig. 7D, E). Moreover, *Pparγ* mRNA was found in the immunoprecipitates of cytosolic GFP-TRIM24^S1043D^ in an RNA immunoprecipitation assay (RIP) (Fig. 7F), and its association with endogenous TRIM24 was also significantly increased when hepatocytes were stimulated with insulin (Fig. 7G). RIP experiments revealed that *Pparγ* mRNA was co-immunoprecipitated with EDC4, and its abundance in the EDC4 immunoprecipitates was increased in the presence of TRIM24, suggesting that TRIM24 might inhibit EDC4-mediated decapping activity to stabilise *Pparγ* mRNA (Fig. 7H). Similarly, *Pparγ* mRNA was also found in the immunoprecipitates of AGO2. However, co-expression of TRIM24 decreased the abundance of *Pparγ* mRNA in the AGO2 immunoprecipitates (Fig. 7I), suggesting that TRIM24 might suppress AGO2 function to stabilise *Pparγ* mRNA. To examine a possible role of P-bodies in regulation of *Pparγ* mRNA, we knocked down crucial components of P-bodies such as LSM1, EDC4, DCP1, AGO1 and AGO2 in wild-type primary hepatocytes via small interfering RNA (siRNA), and found that downregulation of these P-body components all increased *Pparγ* mRNA levels (Supplementary Fig. 10D, H). Similarly to overexpression of TRIM24, silencing EDC4, AGO1 and AGO2 also stabilised *Pparγ* mRNA in cells where transcription was blocked with ActD (Supplementary Fig. 10B, C). Notably, downregulation of EDC4 and AGO2 restored *Pparγ* mRNA levels in primary hepatocytes from the TRIM24^C52/55A^ mice (Fig. 7J–K). Protein expression of P-body components including EDC4, AGOs, LSM1, DCP1 and DDX6 remained normal in the liver of TRIM24^C52/55A^ mice (Supplementary Fig. 9B). However, ubiquitination of EDC4, which inhibited its interaction with DCP1 (Fig. 5E, F), was decreased in the hepatocytes of TRIM24^C52A/C55A^ mice as compared to that in WT cells (Fig. 7L). Together, these data demonstrate that the E3-ligase activity of TRIM24 regulates the stability of *Pparγ* mRNA via the P-bodies. Although insulin represses the translation of *ApoB* mRNA via P-bodies^14^, we observed no alteration of APOB protein in the TRIM24^C52A/C55A^ liver (Supplementary Fig. 9A), suggesting that TRIM24 might not mediate the P-body dependent repression of *ApoB* mRNA translation. Consistently, the expression of microsomal triglyceride transfer protein (MTP), a key factor facilitating primordial lipoprotein assembly and secretion, also remained unchanged in the TRIM24^C52A/C55A^ liver (Supplementary Fig. 9A).

We then investigated whether TRIM24 might regulate *Pparγ* mRNA translation in addition to its stability. To this end, we co-expressed Flag-PPARγ with GFP-TRIM24^S1043D^ or free GFP in cells. When the same amount of Flag-PPARγ plasmid was transfected, co-expression of GFP-TRIM24^S1043D^ increased *Flag*-*Pparγ* mRNA, as compared to co-expression of free GFP, most likely due to the increased stability of *Flag*-*Pparγ* mRNA in the presence of cytosolic GFP-TRIM24^S1043D^ (Supplementary Fig. 11A). Through increasing the Flag-PPARγ plasmid co-transfected with free GFP, the *Flag*-*Pparγ* mRNA could be expressed at the level similar to that of co-expression of GFP-TRIM24^S1043D^ (Supplementary Fig. 11A). The changes of Flag-PPARγ protein paralelled its mRNA when co-expressed with GFP-TRIM24^S1043D^ or free GFP in cells (Supplementary Fig. 11B). We utilised a nascent protein labelling system to examine a possible effect of GFP-TRIM24^S1043D^ on *Flag*-*Pparγ* mRNA translation using free GFP as a control under a condition of comparable *Flag*-*Pparγ* mRNA levels in cells. Under such an experimental condition, GFP-TRIM24^S1043D^ exhibited no apparent effect on the production of nascent Flag-PPARγ in this labelling experiment (Supplementary Fig. 11C, D), suggesting that TRIM24 might not regulate *Pparγ* mRNA translation.

### A TRIM24^S1043A^ knockin mutation attenuates hepatic expression of lipogenic genes and alleviates diet-induced hepatosteatosis

**Fig. 8:**
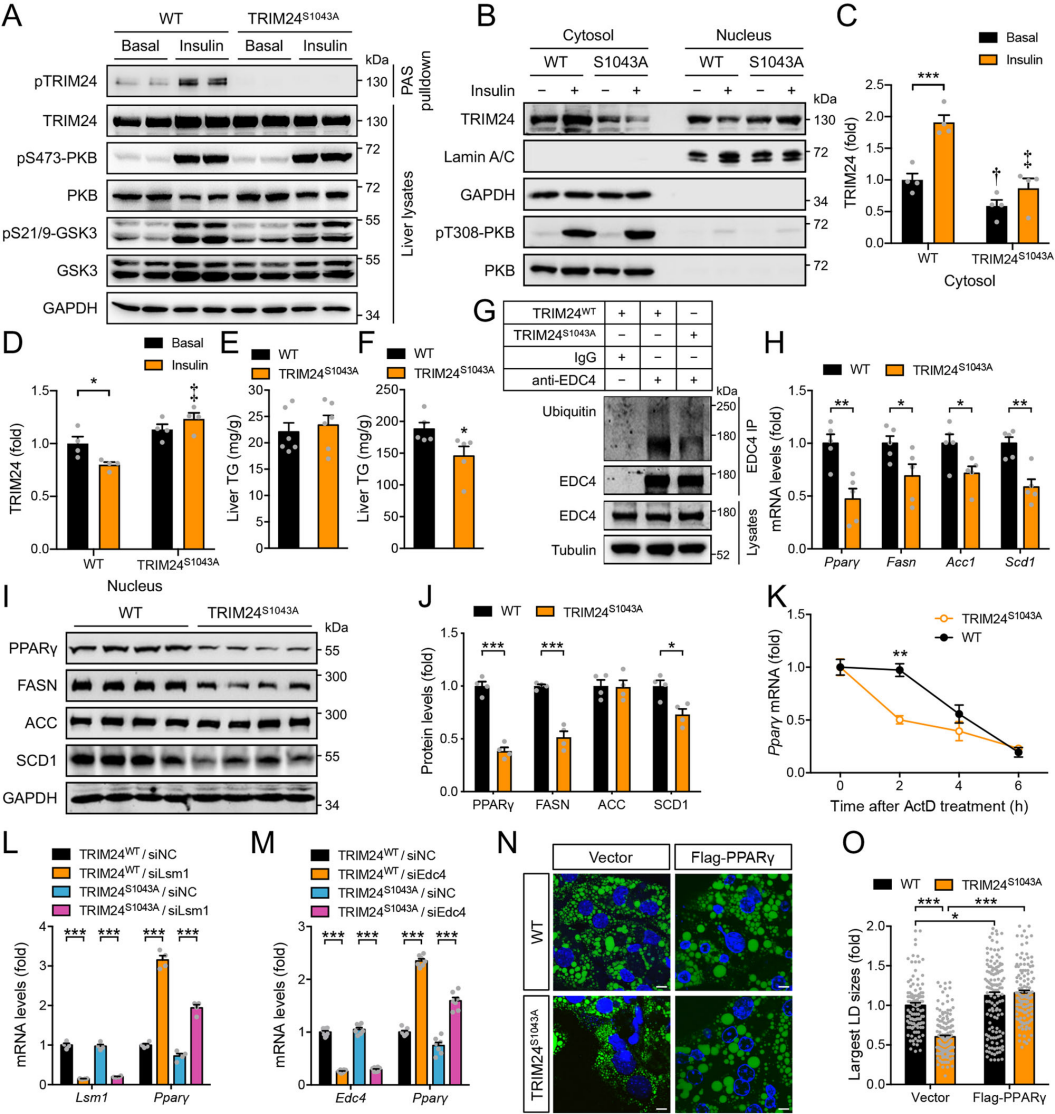
Hepatic lipid metabolism of the TRIM24^S1043A^ mice. **A** Phosphorylation of TRIM24, PKB and GSK3 in 4-month-old female TRIM24^S1043A^ and WT liver upon insulin stimulation. **B**–**D** Subcellular distribution of endogenous TRIM24 in TRIM24^S1043A^ and WT hepatocytes upon insulin stimulation. **B** Representative blots. **C** Quantitation of cytosolic TRIM24. *p* = 0.0002 (WT/Basal vs WT/Insulin). † indicates *p* = 0.031 (WT/Basal vs TRIM24^S1043A^/Basal). ‡ indicates *p* < 0.0001 (WT/Insulin vs TRIM24^S1043A^/Insulin). **D** quantitation of nuclear TRIM24. *p* = 0.018 (WT/Basal vs WT/Insulin). ‡ indicates *p* < 0.0001 (WT/Insulin vs TRIM24^S1043A^/Insulin). *n* = 4. **E**, **F**. Liver TG in 6-month-old male TRIM24^S1043A^ and WT mice on CD (**E**) or HFD (**F**). *n* = 6 (CD) and 5 (HFD). *p* = 0.048. **G** Ubiquitination of endogenous EDC4 in TRIM24^S1043A^ and WT hepatocytes. **H** Expression of lipid metabolic genes in 6-month-old male TRIM24^S1043A^ and WT liver on HFD. *n* = 5. *p* = 0.0037 (*Pparγ*), 0.045 (*Fasn*), 0.030 (*Acc1*) and 0.0022 (*Scd1*). **I**, **J**. PPARγ, FASN, ACC, and SCD1 in 6-month-old male TRIM24^S1043A^ and WT liver on HFD. I, immunoblots. **J** Quantitation data. *n* = 4. *p* = 0.000028 (PPARγ), 0.00017 (FASN) and 0.012 (SCD1). **K** Stability of *Pparγ* mRNA in TRIM24^S1043A^ and WT hepatocytes upon ActD treatment. *n* = 3. *p* = 0.0028 (TRIM24^S1043A^/2 h vs WT/2 h). **L**, **M** *Pparγ* mRNA in TRIM24^S1043A^ and WT hepatocytes upon downregulation of *Lsm1* (**L**), or *Edc4* (**M**). **L** *n* = 4. *p* < 0.0001. **M** *n* = 7 (TRIM24^S1043A^/siNC) and 8 (TRIM24^WT^/siNC, TRIM24^WT^/siEdc4 and TRIM24^S1043A^/siEdc4) for *Edc4*. *n* = 7 for *Pparγ*. *p* < 0.0001. N–O. LDs in TRIM24^S1043A^ and WT hepatocytes transfected with vector or Flag-PPARγ. **N** representative images. **O** quantitation of the largest LDs. Scale bars: 10 μm. *n* = 110 (WT/Vector), 131 (WT/Flag-PPARγ), 164 (TRIM24^S1043A^/Vector) and 122 (TRIM24^S1043A^/Flag-PPARγ). *p* = 0.042 (WT/Vector vs WT/Flag-PPARγ), and *p* < 0.0001 for the rest. Data are given as the mean ± SEM. Statistical analyses: two-way ANOVA for C-D, L-M, O, and two-sided *t*-test for **E**, **F**, **H**, **J**, **K** * indicates *p* < 0.05, **p < 0.01, and *** *p* < 0.001. Source data are provided as a Source Data file.

We next investigated how insulin-stimulated TRIM24-Ser^1043^ phosphorylation regulates P-bodies and hepatic lipogenesis. Protein expression of P-body components including EDC4, AGOs and LSM1 was not altered in insulin-treated hepatocytes (Supplementary Fig. 10I). Expression of GFP-TRIM24 WT, or S1043A and S1043D mutants, did not affect protein levels of EDC4, AGOs and LSM1 (Supplementary Fig. 10J). To investigate the in vivo role of TRIM24-Ser^1043^ phosphorylation in regulating the P-bodies, we generated a TRIM24^S1043A^ knockin mouse model in which the Ser^1043^ on endogenous TRIM24 was mutated to a non-phosphorylatable alanine (Supplementary Fig. 12A, B). The TRIM24^S1043A^ mutant protein was expressed at levels similar to its WT counterpart in various tissues in mice (Supplementary Fig. 12C). PKB-Ser^473^ phosphorylation was comparable in the liver of two genotypes in response to insulin treatment (Fig. 8A). As expected, insulin-stimulated TRIM24 phosphorylation and translocated it into the cytosol in the WT mice, whereas it could not induce corresponding phosphorylation and cytosolic translocation of the mutant TRIM24 in the TRIM24^S1043A^ knockin mice (Fig. 8A–C). Insulin decreased TRIM24 levels in the nucleus of WT hepatocytes but had no such effect in TRIM24^S1043A^ knockin cells (Fig. 8B, D). Similar to the TRIM24^C52A/C55A^ mice, these TRIM24^S1043A^ mice had normal hepatic TG levels when fed with the chow diet but displayed a significant decrease in HFD-induced hepatic TG accumulation, as compared to their WT littermates (Fig. 8E, F). Protein levels of P-body components including EDC4, AGOs, LSM1, DCP1 and DDX6, and another TRIM24 target p53 were unaltered in the liver of TRIM24^S1043A^ mice (Supplementary Fig. 12D, E). Ubiquitination of EDC4 was decreased in the hepatocytes of these mice (Fig. 8G). We then performed gene expression analysis via Q-PCR, and again found decreases of *Pparγ*, *Fasn*, *Acc1* and *Scd1* in the liver of HFD-fed TRIM24^S1043A^ mice (Fig. 8H). Similarly, PPARγ, FASN and SCD1 proteins were lower in the liver of HFD-fed TRIM24^S1043A^ mice than those in the WT liver (Fig. 8I, J). The nuclear run-on experiment showed that transcription of *Pparγ* was increased in the hepatocytes of TRIM24^S1043A^ mice (Supplementary Fig. 12F). However, *Pparγ* mRNA became less stable in TRIM24^S1043A^ hepatocytes than in WT cells (Fig. 8K). In contrast to PPARγ, four other lipogenic transcription factors, namely SREBP1, ChREBP1, LXRα and USF1, were unaltered at the mRNA level (Supplementary Fig. 12G), and flSREBP1, nSREBP1 and ChREBP1 proteins were also normal in the TRIM24^S1043A^ liver (Supplementary Fig. 12E). Furthermore, the key regulators of fatty acid oxidation, PPARα and CPT1, and the primary apolipoprotein APOB and the lipoprotein assembly regulator MTP, were all normal in the TRIM24^S1043A^ liver (Supplementary Fig. 12E). To establish a causal relationship between the P-bodies and *Pparγ* expression in these mice, we isolated primary hepatocytes and silenced LSM1 or EDC4 in these cells. Importantly, downregulation of LSM1 or EDC4 both could rescue *Pparγ* expression in TRIM24^S1043A^ primary hepatocytes (Fig. 8L, M), showing that the P-bodies are responsible for the diminution of *Pparγ* expression. Like TRIM24^C52/55A^ primary hepatocytes, TRIM24^S1043A^ primary hepatocytes also contained smaller LDs than WT cells, which was rescued by overexpression of PPARγ (Fig. 8N, O).

These data demonstrate the importance of TRIM24-Ser^1043^ phosphorylation in the regulation of hepatic *Pparγ* expression through the P-bodies, and its inhibition alleviates of HFD-induced hepatosteatosis.

## Discussion

**Fig. 9:**
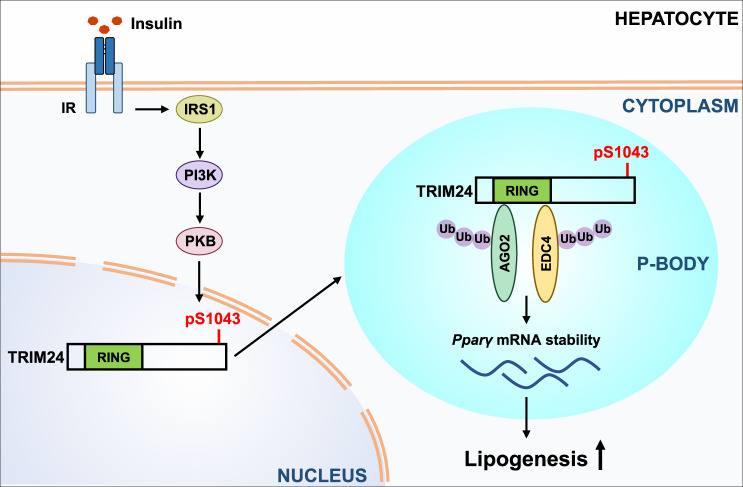
A working model of TRIM24 in regulation of P-bodies in response to insulin. A model for the insulin–PKB–TRIM24 signalling pathway in control of hepatic lipogenesis via P-bodies. Insulin stimulates phosphorylation of TRIM24 via PKB in hepatocytes, which translocates TRIM24 from the nucleus into the cytoplasm. Cytosolic TRIM24 is targeted to *Pparγ*-containing P-bodies, where it interacts with P-body components such as EDC4 and AGO1/2 and ubiquitinates these proteins to stabilise *Pparγ* mRNA. This pathway promotes lipid accumulation in the liver, and its inhibition alleviates diet-induced hepatosteatosis.

Our findings shed light on how insulin signalling regulates mRNA storage/degradation via P-bodies in the liver. We propose a model in which the insulin-activated PKB/Akt phosphorylates TRIM24, which consequently shuttles from the nucleus into the cytoplasm to regulate *Pparγ*-containing P-bodies (Fig. 9). This pathway, in turn, regulates the stability of *Pparγ* mRNA, and lipid accumulation in the liver.

It has been well documented in the literature that insulin regulates mRNA transcription and translation^2^. Our study has revealed a previously unrecognised molecular link between insulin signalling and control of mRNA stability by P-bodies, which increases the complexity of regulation of gene expression by insulin. P-bodies are membrane-less organelles with diverse functions to translationally repress, store or degrade mRNAs^9,10^. Our data identify TRIM24 as a regulatory factor that dynamically associates with P-bodies. However, only a portion of foci of EDC4, AGO2, and LSM1 colocalise with cytosolic TRIM24 in hepatocytes, suggesting the presence of heterogeneity/specificity of P-body populations. We name these TRIM24-associated P-bodies as TAP-bodies, which may represent a sub-population of P-bodies. It remains unclear how TRIM24 is targeted to TAP-bodies when it translocates into the cytosol upon stimulation with insulin. Nevertheless, this finding demonstrates that P-bodies are heterogeneous and may be differentially specifically regulated, presumably in response to different stimuli. Types of sequestrated mRNAs may differ in sub-populations of P-bodies. It would be intriguing to have a thorough profile of mRNAs associated with TAP-bodies, which may help to delineate the specific cellular functions of this type of P-bodies. Another exciting aspect of TRIM24 being a component of P-bodies is its dynamic association with this organelle. Phosphorylation of TRIM24 by the insulin−PKB pathway represents a crucial step for dynamic regulation of the sub-population of P-bodies, TAP-bodies. TRIM24 interacts with multiple components of P-bodies such as EDC4, AGOs, and LSM1, which display distinct functions in mRNA regulation. Our data suggest that TRIM24 ubiquitinates EDC4 through chain linkages of K6, K27, K29 and K33 to inactivate the decapping enzyme complex to stabilise *Pparγ* mRNA. It ubiquitinates AGO2 mainly through chain linkages of K27, K29 and K33, which also results in the stabilisation of *Pparγ* mRNA. Whether and how TRIM24 ubiquitinates other components in P-bodies remains unclear, which deserves investigations in future. Although our data show that the decreased stability of *Pparγ* mRNA may be the main mechanism underlying the phenotypic effects on hepatosteatosis in the TRIM24^C52/55A^ and TRIM24^S1043A^ mice, it is still possible that some unknown mechanisms might also contribute to the phenotypic changes in these mice. Moreover, TRIM24 has also been linked to regulation of glucose metabolism in cancer cells^27,28^. Therefore, a thorough investigation of potential ubiquitination substrates of TRIM24 as well as its mRNA targets at both transcriptional and post-transcriptional levels is needed to fully delineate the metabolic function of TRIM24.

TRIM24 is a multi-functional protein acting as an E3-ligase^20^, a co-regulator of nuclear receptors^17,18^, and a histone “reader”^19^. Whole-body loss of TRIM24 down-regulates lipogenic gene expression in the liver partly through its function as an epigenetic co-regulator of transcription^29^. TRIM24 deficiency causes a decrease in overall hepatic TG levels but an accumulation of lipids in lesions in the liver^29^. The TRIM24^C52/55A^ mice had normal hepatic TG levels when fed on a chow diet, whereas they had diminutions in their hepatic lipogenic genes and TG accumulation when fed on HFD. The similarity and difference between these two mouse models might reflect the involvements of different functional domains of TRIM24 in the regulation of hepatic lipid metabolism. Retention of all functional domains of TRIM24 in the nucleus through the TRIM24^S1043A^ mutation causes effects on hepatic TG similar to the TRIM24^C52/55A^ mutation, suggesting a deficiency of the E3-ligase activity of TRIM24 in the cytosol might underlie the liver phenotype. Thus, shuttling of TRIM24 from the nucleus into the cytoplasm upon insulin stimulation provides a potential point to intervene and restrict its pro-lipogenic activity in the cytoplasm. HFD causes chronic hypersecretion of insulin that increases hepatic lipogenesis through multiple transcription factors such as USF, SREBP-1c, LXRα and ChREBP1^5^. Expression of the early gene 4 open reading frame-1 (E4orf1) derived from human adenovirus decreases hyperinsulinemia in HFD-fed mice and alleviates hepatosteatosis^30^. Moreover, the disruption of insulin signalling through overexpressing an mTOR-defective CRTC2 mutant inhibits SREBP1 processing thereby mitigating hepatosteatosis^31^. Therefore, our study is in line with these reports, suggesting that targeting hyperinsulinemia or insulin signalling on hepatic lipogenesis might be of therapeutic value to treat hepatosteatosis.

Identifying TRIM24 as a mechanistic link between insulin signalling and hepatic lipid metabolism highlights this protein as a potential target for developing drugs to treat hepatosteatosis. As a proof of concept, our study demonstrates that the E3-ligase of TRIM24 may be targeted to treat hepatosteatosis at least partially by regulating *Pparγ* mRNA stability likely via the P-bodies. Our results are in agreement with a steatogenic role of hepatic PPARγ that is up-regulated during HFD feeding^32^ and whose disruption in the liver alleviates hepatosteatosis in *ob*/*ob* mice^33^ and HFD-fed mice^25^. However, a caution from recent studies is that TRIM24 can function as either a tumour suppressor or an oncogene in a cancer-type dependent manner^17,19,27,34,35^. The tumour-related functions of TRIM24 are linked to its role as a transcriptional co-regulator in the nucleus. For instance, TRIM24 deficiency confers an oncogenic activity by relieving its inhibitory effect on the RARα^17^. The PHD-Bromo domain of TRIM24 can bind to histones through combinatorial recognition of unmethylated H3K4 and acetylated H3K23 within a single histone tail, which, together with the binding of TRIM24 to the oestrogen receptor, activates a subset of oestrogen-dependent genes^19^. Overexpression of TRIM24 negatively correlates with survival of breast cancer patients^19^. Our data demonstrate that control of hepatic lipid accumulation is related to the E3-ligase activity of TRIM24 that regulates EDC4 and AGO2. Therefore, inactivation of the E3-ligase activity of TRIM24 might not confer the oncogenic activity that TRIM24 deficiency does. Nevertheless, evaluation needs to be carried out in the future regarding the potential effects of the TRIM24^C52/55A^ knockin mutation on tumorigenesis.

TRIM24 belongs to a small protein family containing two other members, TRIM28 and TRIM33^36^. All three proteins are transcriptional co-regulators with E3-ligase activities. The E3-ligase of TRIM33 plays a critical role in cytosolic RNA-induced NLRP3 inflammasome activation through ubiquitination of DExD/H-box helicase DHX33 in the cytoplasm^37^. Interestingly, a phosphorylation site (flanking sequence, PRRKRLK**s**DERPVHI, Ser^1119^ in lower case identified as a phosphorylated residue (www.phosphosite.org)) is present in the predicted monopartite NLS of TRIM33 (IQPRRKRLKSD, www.NLS-mapper.lab.keio.ac.jp). We could speculate that Ser^1119^ phosphorylation of TRIM33 might also causes this protein to shuttle from the nucleus into the cytoplasm to regulate innate immunity. Unlike TRIM24 and TRIM33, TRIM28 has a predicted bipartite NLS (469-KRSRSGEGEVSGLMRKVPRVS-489) in its middle region but contains no paralogous phosphorylation site at its C-terminus. We suspect that these three proteins might be differentially regulated in respect to their subcellular localisation. Our data suggest that the NLS2 on TRIM24 might have dual functions in nuclear import and export with Ser^1043^ phosphorylation serving as a switch between these two modes. However, we are currently unable to fully rule out the possibility that Ser^1043^ phosphorylation might impair nuclear import of TRIM24 in addition to its function to promote nuclear export.

In summary, our findings demonstrate that TRIM24 is a critical regulator of P-bodies linking insulin signalling to mRNA stability. Its E3-ligase activity is a potential target for drug discovery to combat hepatosteatosis.

## Methods

This study was approved by the Ethics Committee of Nanjing University complying with all relevant ethical regulations. All mouse procedures in this study were carried out under approval of the Institutional Animal Care and Use Committee (IACUC) at Model Animal Research Center of Nanjing University with the protocol number MARC-CS24.

### Materials

Recombinant human insulin was purchased from Novo Nordisk (Bagsvaerd, Denmark). Akti1/2 was from Merck Millipore (Darmstadt, Germany), and PI-103 from Enzo Life Sciences (Farmingdale, NY, USA). Protein G-Sepharose was from GE Healthcare (Buckinghamshire, UK). High-fat diet (60 kcal% fat, Cat. No. 12492) was purchased from Research Diets (USA). All other chemicals were from Sigma-Aldrich (Shanghai, China) or Sangon Biotech (Shanghai, China). The commercial antibodies and resins used are listed in Supplementary Table 1. For immunoblotting, the commercial antibodies were used at 1:1000 dilution. The antibody recognising pSer^1043^-TRIM24 was generated at ABclonal (Wuhan, China) and used at 1 μg/ml.

### Molecular biology

Human TRIM24 (NP_056989.2), mouse TRIM24 (NP_659542.3), human EDC4 (NP_055144.3), human AGO1 (NP_036331.1), human AGO2 (NP_036286.2), human LSM1 (NP_055277.1), human DCP1 (NP_001277134.1), and mouse PPARγ (NP_035276.2) cDNAs were cloned into mammalian expression vectors pcDNA5-FRT/TO-GFP, pcDNA5-FRT/TO-HA, pcDNA5-FRT/TO-Flag, or pcDNA5-FRT/TO-Myc. Site-directed mutagenesis was carried out using standard procedures. Human TRIM24 cDNA was cloned into the bacterial expression vector pGEX6P for the production of GST-TRIM24 fusion protein in *Escherichia coli*. All plasmids were sequenced at Life Technologies (Shanghai, China).

### Generation of the TRIM24^C52/55A^ and TRIM24^S1043A^ knockin mice

TRIM24^C52/55A^ and TRIM24^S1043A^ knockin mice were generated on a C57Bl/6J background using the CRISPR/Cas9-based strategy outlined in Supplementary Fig. 8A and Supplementary Fig. 12A by the transgenic facility at Nanjing University and GemPharmatech Co. Ltd, respectively, and are readily available from Drs. Hong-Yu Wang and Shuai Chen. To generate TRIM24^C52/55A^ knockin mice, the cysteine residues Cys^52^/Cys^55^ (the surrounding sequence is LLDT**c**AV**c**HQNIQ, Cys^52^/ Cys^55^ shown in lower case bold) on TRIM24 were mutated to alanine through CRISPR/Cas9-mediated knockin mutagenesis. A *Pst*I enzyme restriction site was also introduced to facilitate genotyping. The TRIM24^C52/55A^ knockin mice were genotyped via amplification of the mutated region (676 bp) using two primers (5′-GTTGACAGCTCCGCGTCTTC-3′ and 5′-AGCAGCTAACTCGCCACAACC-3′), followed by restriction digestion with *Pst*I (329/347 bp cleaved products for TRIM24^C52/55A^ knockins). For generation of TRIM24^S1043A^ knockin mice, the Ser^1043^ (the surrounding sequence is QPRKKRLK**s**TEDRQLL, Ser^1043^ shown in lower case bold) on TRIM24 was substituted to alanine through CRISPR/Cas9-mediated mutagenesis. A *Bpu*EI enzyme restriction site in WT allele was removed through synonymous mutations to facilitate genotyping. The mutated region (728 bp) was amplified using two primers (5′-CACTCCTTCTGGAAGGAGTCAGTAC-3′ and 5′-TCTTAGTGTAGCACTCACTCCAGG-3′), and digested with *Bpu*EI to generate 361/367 bp cleaved products for WTs and 728 bp non-cleaved products for TRIM24^S1043A^ knockins.

### Mouse breeding and husbandry

Mice were housed in an animal facility free of specific pathogens with a light/dark cycle of 12 h, ambient temperature 23 ± 2 °C and humidity 40–70%, and had free access to food and water unless stated. Mating of male heterozygotes with female heterozygotes was set up to generate homozygous knockin mice and WT littermates that were used in experiments. The TRIM24^C52/55A^ knockin mice and their WT littermates were fed with HFD for 3 months, and the TRIM24^S1043A^ knockin mice and their WT littermates were fed with HFD for 4 months, before sacrificed for analyses.

### Serum and liver TG

Measurement of serum TG was performed using the Wako LabAssay Triglyceride kit (290-63701) (Wako Chemicals USA). Liver TG was determined through the measurement of its glycerol contents^38^. Briefly, liver tissue chunks were subjected to saponification in ethanolic KOH, and the released free glycerol from TG was measured with the Free Glycerol Reagent (F6428, Sigma-Aldrich).

### Cell culture, transfection, stimulation and lysis

Human embryonic kidney HEK293 cells (1101HUM-PUMC000010) were obtained from the Cell Resource Center, Chinese Academy of Medical Sciences and Peking Union Medical College (China), and maintained in medium containing 10% (v/v) foetal bovine serum. Isolation and culture of primary mouse hepatocytes were carried out as previously described^39^. Cell transfection was carried out using a Lipofectamine-3000 (Thermo Fisher Scientific) mediated method as previously described^40^. Small interfering RNAs (siRNAs) for targeting mouse genes in the study are as follows. siEdc4: 5′-GCGCGUGCUUUAGUUCUAUTT-3′. siLsm1: 5′-CCUGGUCACCUCACACAUUTT-3′. siAgo1: 5′-GCUUCUGGCCAAUUACUUUTT-3′. siAgo2: 5′-GCACAUGGUCCAGCACUUUTT-3′. siDcp1: 5′-GGGAGAUGCAUCACAGAAATT-3′.

Cells were deprived of serum for 16 h (basal), and then stimulated with insulin (100 nM) for 30 min. As for treatments with inhibitors, cells were incubated with PI-103 (10 µM) or Akti1/2 (10 µM) for 30 min prior to stimulation with insulin. For inhibition of nuclear export, cells were pre-treated leptomycin B (100 ng/ml) for 60 min. After removal of culture media, cells were washed and lysed in a lysis buffer (50 mM Tris-HCl (pH 7.4), 1 mM EDTA, 1 mM EGTA, 1 mM sodium *o-*vanadate, 10 mM sodium glycerophosphate, 50 mM NaF, 5 mM sodium pyrophosphate, 0.27 M sucrose, 2 µM microcystin-LR, 1 mM benzamidine, 0.1% (v/v) 2-ME, complete proteinase inhibitor mixture (Roche) and 1% (w/v) Triton X-100).

### Proximity-dependent biotin identification (BioID) assay

BioID assay was carried out as previously described^41^. Briefly, HEK293 cells stably expressing Myc-BirA*-EDC4 were transiently transfected with Free-GFP or GFP-TRIM24. After transient transfection, culture media were supplemented with 50 mM Biotin for 24 h. After harvest, cell lysates were incubated with NeutrAvidin-Sepharose beads at 4 °C overnight. After removal of non-specific binding proteins, biotinylated proteins were eluted in sample buffer and subjected to immunoblotting analysis using specific antibodies.

### RNA immunoprecipitation (RIP) assay

RIP assay for detection of protein-RNA interactions was performed in mouse primary hepatocytes and HEK293 cells as previously described with modifications^42^. Briefly, cells were treated with 1% formaldehyde at room temperature for 10 min to cross-link protein-RNA complexes. Afterwards, glycine was added into the media to a final concentration of 125 mM, and cells were further incubated with gentle shaking for 5 min at room temperature. After rinsed with cold PBS, cells were lysed in RIP buffer (150 mM KCl, 25 mM Tris-Cl (pH = 7.4), 5 mM EDTA, 0.5 mM DTT, 0.5% NP40, 100 U/ml RNAase inhibitor, protease inhibitor cocktail (MCE)). Cell lysates were incubated with antibody-coupled protein G Sepharose at 4 °C overnight to immunoprecipitate protein-RNA complexes. Immunoprecipitates were washed with RIP buffer to remove non-specific binding proteins, and co-immunoprecipitated RNAs were isolated by resuspending beads in TRIzol® Reagent (Life Technologies) according to manufacturer’s instructions.

### Subcellular fractionation

Subcellular fractionation was carried out to obtain nuclear and cytosolic fractions using NE-PER^TM^ Nuclear and Cytoplasmic Extraction Reagents (ThermoFisher Scientific, Cat No. #78833). Briefly, after detaching from plates with trypsin, cells were collected via centrifugation at 500 × *g* for 5 min, and washed with PBS. Afterwards, CER I:CER II:NER reagents (volume ratio, 200:11:100) were added to cells that were pelleted. Cell pellets were then vigorously resuspended by vortexing. Cell suspensions were incubated on ice for 10 min, and added to ice-cold CERII. After vortexing and incubating on ice for 1 min, cell lysates were centrifuged at 16,000 × *g* for 5 min. The resultant supernatants (cytosolic extracts) were transferred into a clean pre-chilled tube. The resultant pellets containing nuclei were resuspended in ice-cold NER with the same volume as the cytosolic extracts. The suspensions were placed on ice for 40 min, and vortexed for 15 sec at 10-min intervals. The samples were then centrifuged at 16,000 × *g* for 10 min, and the resultant supernatants (nuclear extracts) were transferred into a clean pre-chilled tube. Equal volumes of cytosolic and nuclear fractions were subjected to immunoblotting analysis.

### Tissue lysis, and protein measurement

Mouse tissues were harvested and homogenised in the lysis buffer using a Polytron homogeniser (Kinematica, Luzern, Switzerland). Tissue debris was removed via centrifugation, and protein concentrations of resultant homogenates were determined using Bradford reagent (Thermo Fisher Scientific).

### Immunoprecipitation

Immunoprecipitation was performed as previously described^43^. Briefly, cell lysates were incubated with the indicated resins overnight at 4 °C. The resins were washed to remove non-specific binding proteins. Afterwards, the immunoprecipitates were denatured in SDS sample buffer and eluted from the resins for subsequent analysis.

### Immunoblotting

After electrophoretic separation on SDS-PAGE gels, proteins were immunoblotted onto nitrocellulose membranes. After blocking with milk, membranes were sequentially incubated with the indicated primary antibodies and horseradish-peroxidase-conjugated secondary antibodies. Membranes were incubated with ECL® (enhanced chemiluminescence reagent; GE Healthcare), and signals were detected using a gel documentation system (Syngene, UK). Each lane in immunoblots represents a biological replicate. Chemiluminescent signals were quantified using the Image J (version 1.46). Signal intensities of proteins of interest were normalised with loading controls and presented as fold changes. Phosphorylation of TRIM24 was normalised with its total protein and presented as fold changes.

### Nascent protein assay

HEK293 cells were transfected with Flag-PPARγ in combination with GFP-TRIM24^S1043D^ or free GFP for 48 h. Afterwards, nascent proteins were labelled using Click-IT^TM^ AHA (L-azidohomoalaine) from Thermo Fisher Scientific for indicated time. Cells were lysed and total Flag-PPARγ was immunoprecipitated from cell lysates. The immunoprecipitated Flag-PPARγ was reacted with Click-IT^TM^ Protein Reaction Buffer Kit. After labelling, the nascent Flag-PPARγ in the immunoprecipitates was detected via immunoblotting using the HRP-labelled Avidin antibody. Immunoblotting signals of nascent and total Flag-PPARγ in the immunoprecipitates were quantified using ImageJ. Nascent Flag-PPARγ was normalised with total Flag-PPARγ and presented as fold changes.

### Mass spectrometry

After immunoprecipitation, protein eluates were separated in precast NuPAGE^®^ Bis-Tris gels via electrophoresis. Protein bands stained with Coomassie dye were excised, destained and digested with trypsin. The peptides resulting from trypsin digestion were analysed by LC–MS/MS in which a NanoLC.2D (Eksigent Technologies) was coupled with a TripleTOF 5600+ System (AB SCIEX) for acquisition of MS data. Original MS/MS data were analysed via ProteinPilot Software (version 4.5, AB Sciex), and corresponding peptides and proteins were identified via search against UniProt database.

### LD staining

For quantitative analysis of LDs, primary hepatocytes were transfected with Flag-PPARγ or Flag vector for 48 h, then fixed and stained with Bodipy 493/503. Images were taken with a Leica SP5 confocal microscope. Sizes of the largest LD in each cell were measured using Image J software.

### RNA fluorescence in situ hybridisation (RNA FISH)

Cy3-labelled *Pparγ* probes were obtained from RiboBio (Guangzhou,China). RNA FISH was performed using fluorescent in situ hybridisation kit following the manufacturer’s instructions. Briefly, cells overexpressing different proteins were rinsed in PBS and fixed in 4% formaldehyde for 10 min. Cells were then permeabilized in PBS containing 0.5% Triton X-100 for 5 min, washed with PBS three times for 5 min, and pre-hybridised at 37 °C for 30 min before hybridisation. Then an anti-*Pparγ* oligodeoxynucleotide probe was used in the hybridisation solution at 37 °C overnight in the dark. On the next day, cells were counterstained with DAPI and imaged using a confocal laser-scanning microscope (Carl Zeiss LSM880).

### Global nuclear run-on assay

A global nuclear run-on assay was performed in mouse primary hepatocytes as previously described^44^. Briefly, 1 × 10^7^ nuclei were isolated and used for each run-on reaction. 3 biological replicates were produced. Br-UTP was incorporated into on-going transcription by run-on reaction which was performed at 30 °C for 7 min. Total RNA was extracted with TRIzol® Reagent (Life Technologies) and fragmented with RNA Fragmentation Reagent (Life Technologies). Fragmented RNA was purified, and then treated by T4 polynucleotide kinase (PNK; New England Biolabs) to dephosphorylate the 3’end of RNA fragments. Br-UTP labelled RNA was enriched twice with anti-BrdU beads (Santa Cruz Biotech) and precipitated overnight. Poly(A) tailing was done using an *E. coli* Poly(A) Polymerase (New England Biolabs). Processed RNA was used for reverse-transcription into cDNA with a PrimeScript® RT reagent kit (TaKaRa). Expression levels of target genes were determined via real-time Quantitative PCR using an Applied Biosystems® StepOnePlus^TM^ system.

### Confocal microscopy

Confocal microscopy was carried out as previously described^40^. Briefly, transfected cells expressing fluorescent fusion proteins were stained with 4′,6-diamidino-2-phenylindole (DAPI) and fixed with 4% paraformaldehyde. Flag-tagged proteins were sequentially stained with anti-Flag antibody and Cy3-conjugated secondary antibody. Slides were mounted and photographed using a Leica or Olympus confocal microscope.

### Real-time quantitative PCR

Total RNA was isolated from mouse tissues, cells or RIP assay using the TRIzol® Reagent (Life Technologies) and used for reverse-transcription into cDNA with a PrimeScript® RT reagent kit (DRR047A, TaKaRa). Expression levels of target genes were determined via real-time Quantitative PCR using an Applied Biosystems® StepOnePlus^TM^ system. The primers for Quantitative PCR were listed in Supplementary Table 2.

### Statistics and reproducibility

Data are given as the mean ± SEM. Statistical analyses were performed via *t*-test for two groups or via two-way ANOVA for multiple groups using Prism software (version 9.0, GraphPad, San Diego, CA, USA). Differences were considered statistically significant at *P* < 0.05.

Similar results were obtained from at least two independent experiments for Figs. 1A, B, D–F; 3A, C, D, E–H; 4A, G–J; 5A, C–E, G–K; 6A, B; 7D, E, L; 8A and Supplementary Figs. 2A–G; 3A–F; 4A–C; 5; 6A, B; 7A, B, D; 8J, K; 9I, J; 10B, C; 11C–E.

### Reporting summary

Further information on research design is available in the Nature Research Reporting Summary linked to this article.

## Supplementary information

Supplementary Information

Description of Additional Supplementary Files

Supplementary Data 1

Supplementary Data 2

Supplementary Data 3

Reporting Summary

## Data Availability

The proteomic data generated in this study have been deposited in the MassIVE Repository (University of California, San Diego) under accession codes “MSV000087849 [https://massive.ucsd.edu/ProteoSAFe/dataset.jsp?task=bd274a579b0e48a1b826174ec2b22915]” and “MSV000087850 [https://massive.ucsd.edu/ProteoSAFe/dataset.jsp?task=c22ee602b9ad4cb79ca89bd977ef26a5]”. The RNA-Seq data generated in this study have been deposited in the NCBI database under accession code “GSE193774”. All other relevant data supporting the key findings of this study are available within the article and its Supplementary Information files or from the corresponding author upon reasonable request. Source data are provided with this paper.

## Source data

Source data
