# Supplementary material for: TRIM24 is an insulin-responsive regulator of P-bodies
Source: Nat Commun. 2022 Jul 8;13:3972. doi: 10.1038/s41467-022-31735-0 (PMC9270398; doi:10.1038/s41467-022-31735-0)
Supplement: Supplementary file 1 — Supplementary Information [file 41467_2022_31735_MOESM1_ESM.pdf]

Supplementary Information for

TRIM24 is an insulin-responsive regulator of P-bodies

Wei et al.

Supplementary Table 1 The list of antibodies used in this study

The commercial primary antibodies were used at a dilution of 1:1000 for immunoblotting.

**Antibody Name**	**Company**	**Cat No.**
Rabbit anti-TRIM24	Proteintech	14208
Mouse anti-GAPDH	proteintech	60004-1-lg
Mouse anti-GFP	Santa Cruz Biotechnology	sc-9996
Mouse anti-c-Myc	Santa Cruz Biotechnology	sc-40
Mouse anti-EDC4	Santa Cruz Biotechnology	sc-376382
Mouse anti-eIF2C/AGO1-4	Santa Cruz Biotechnology	sc-376696
Mouse anti-LSM1	Santa Cruz Biotechnology	sc-373685
Mouse anti-GW182	Santa Cruz Biotechnology	sc-56314
Mouse anti-SCD1	Santa Cruz Biotechnology	sc-81776
Mouse anti- DCP1	Santa Cruz Biotechnology	sc-100706
Mouse anti-RCK	Santa Cruz Biotechnology	sc-376433
Rabbit anti-SREBP1	Santa Cruz Biotechnology	sc13551
Rabbit anti-PPAR α	Santa Cruz Biotechnology	sc-9000
Anti-Flag M2 Affinity Gel	Sigma	A2220
Rabbit anti-Tubulin	Bioword	BS1699
Rabbit anti-Lamin A/C	Abclonal	A0249
Rabbit anti-FASN	Cell Signaling Technology	3189
Rabbit anti-ACC	Cell Signaling Technology	3676
Rabbit anti-PPARγ	Cell Signaling Technology	2435
Rabbit anti-HA	Cell Signaling Technology	# 3724S
Rabbit anti-PKB	Cell Signaling Technology	#9272
Rabbit anti-pS473-PKB	Cell Signaling Technology	#9271
Rabbit anti-pT308-PKB	Cell Signaling Technology	3038
phospho-Akt substrate (PAS) antibody	Cell Signaling Technology	#9611
Mouse monoclonal anti-MTP	BD Transduction LaboratoriesTM	612022
PAS Sepharose beads	Cell Signaling Technology	#9646
GFP-Trap®-agarose	Chromotek	gta-10

Supplementary Table 2 Primer information for QPCR analysis of expression of target genes

Gene name	Forward primer	Reverse primer
actin	5'-GAGAGGGAAATCGTGCGTGACA-3'	5'-GTTTCATGGATGCCACAGGAT-3'
Acc1	5’-TGATGTGGTGGTCCACTCTGA-3’	GGCCAACGGAGATGGTTCAT
Ago1	5’-GGAGGTGGTGGAATACATGG-3’	5'-GGCTAGCCACTTGATGGAGA-3’
Ago2	5’-ATGGACATCCCCAAAATTGA-3’	5’-GATGCGATCTTTGCCTTCTC-3’
Dcp1a	5’-GCCTTACCATGGCTTTACCA-3’	5'-CGCCGTGTCTCCTCTTCTAC-3’
Edc4	5’-AGCATCTGCGGGACATACTC-3’	5'-CTCCAGAGAGGCAAATGACC-3’
Fasn	5’-GCTTCGCCAACTCTACCATG-3’	5’-CCATCGCTTCCAGGACAATG-3’
Lsm1	5’-TCATCGAGGACATCGACAAA-3’	5'-GCGTGTCACTCTCCTTCTCC-3’
Pparγ	5’-TCGCTGATGCACTGCCTATG-3’	5’-GAGAGGTCCACAGAGCTGATT-3’
Scd1	5’-ATGTCTGACCTGAAAGCCGA-3’	5’-GAAGGTGCTAACGAACAGGC-3’
Srebp1	5’-TCAGCAGCCCCTAGAACAAA-3’	5’-CTGATGCCTGCAGTCTTCAC-3’
Chrebp1	5’-CCCTCAGACACCCACATCTT-3’	5’-TCAGAAAGGGGTTGGGATCC-3’
Lxrα	5’-GCATGATCGAGAAGCTGGTG-3’	5’-GTCTTCAGCAAGGCGATCTG-3’
Usf1	5’-CCACCCTTATTCCCCGAAGT-3’	5’-TCCACCTTTACTCTGGCCAG-3’
Trim24	5’-CTTGCAAAGGACCATCGAAT-3’	5'-TCACACCTTGCACGAAGAAG-3’
36b4	5’-ATCCCTGACGCACCGCCGTGA-3’	5’-TGCATCTGCTTGGAGCCCACGT-3’

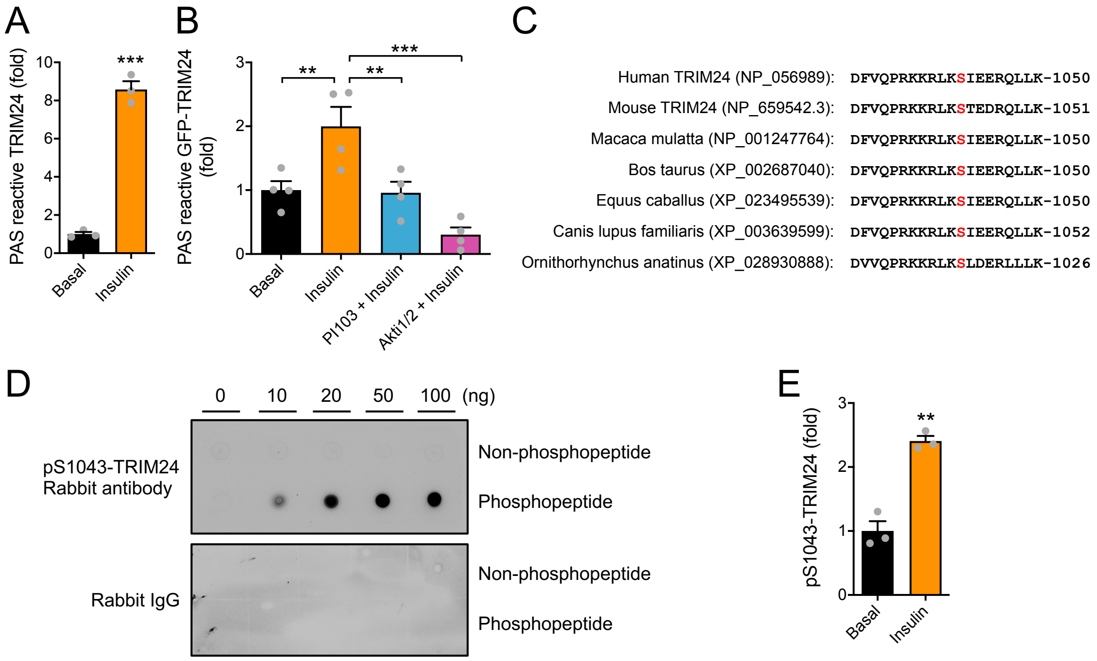

Supplementary Figure 1 Insulin-induced phosphorylation of TRIM24

A. PAS-reactive phosphorylation of TRIM24 in mouse liver in response to insulin. Phosphorylated proteins recognised by the PAS antibody were pulled down from lysates of mouse liver treated with or without insulin using a PAS antibody-conjugated resin. TRIM24 was detected in the immunoprecipitates via western blot using the specific antibodies. Immunoblots were shown in Fig. 1D. n = 3. $p = 6.81e-5$.

B. Effects of inhibitors of PI-3K and PKB on insulin-induced PAS-reactive phosphorylation of TRIM24. GFP-TRIM24 was expressed in HEK293 cells stimulated with or without insulin after pre-treatment with a PI-3K inhibitor PI103, or a PKB inhibitor Akti1/2, or vehicle. After immunoprecipitated from cell lysates, phosphorylation of TRIM24 was determined using the PAS antibody. Immunoblots were shown in Fig. 1E. n = 4. $p = 0.0039$ (Insulin vs Basal), 0.0030 (Insulin vs PI103+Insulin), and $p < 0.0001$ (Insulin vs Akti1/2+Insulin).

C. Sequence alignment of the PAS-reactive phosphorylation site of TRIM24 proteins from different mammals.

D. Specificity of pS1043-TRIM24 antibody. The pS1043-TRIM24 phosphopeptide and corresponding non-phosphopeptide were spotted onto the nitrocellulose membrane with indicated amounts. The pS1043-TRIM24 antibody and a rabbit IgG were used for detecting the phosphopeptide.

E. Ser^{1043} phosphorylation on endogenous TRIM24 in response to insulin. Mouse primary hepatocytes were stimulated with or without insulin. Endogenous TRIM24 was immunoprecipitated from cell lysates and Ser^{1043} phosphorylation on it was determined using the site-specific phospho-antibody. Immunoblots were shown in Fig. 3F. n = 3. $p = 0.0012$.

Data are given as the mean ± SEM. Statistical analyses were carried out via two-sided t-test for A and E, and one-way ANOVA for B. ** indicates $p < 0.01$, and *** indicates $p < 0.001$. Source data are provided as a Source Data file.

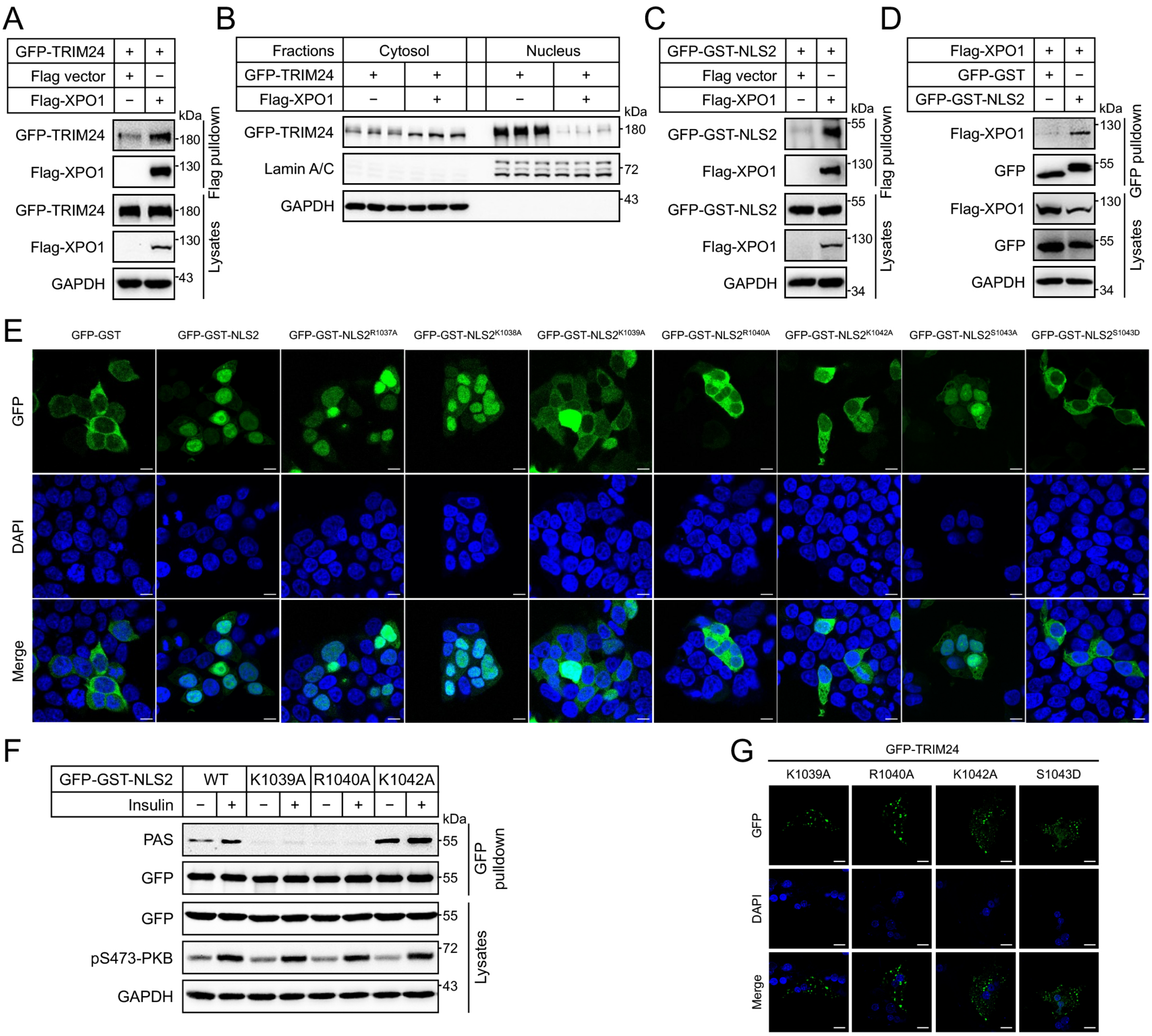

Supplementary Figure 2 Nuclear import and export of TRIM24

A. Interaction of Flag-XPO1 and GFP-TRIM24 that were co-expressed in HEK293 cells. Immunoprecipitation was performed using the Flag antibody.

B. Subcellular distribution of GFP-TRIM24 in the presence or absence of Flag-XPO1 in HEK293 cells. GFP-TRIM24 subcellular distribution was determined in the nuclear and cytosolic fractions via immunoblotting.

C. Interaction of Flag-XPO1 and GFP-GST-NLS2 that were co-expressed in HEK293 cells. Immunoprecipitation was performed using the Flag antibody.

D. Interaction of Flag-XPO1 and GFP-GST-NLS2 that were co-expressed in HEK293 cells. Immunoprecipitation was performed using the GFP-Trap resin.

E. Subcellular localisation of GFP-GST-NLS2 WT and mutant proteins in HEK293 cells. Scale bars indicate 10 μm in length.

F. PAS-reactive phosphorylation of GFP-GST-NLS2 WT and mutants in HEK293 cells in response to insulin. PAS-reactive phosphorylation was detected on the immunoprecipitated GFP-GST-NLS2 WT and mutants via immunoblotting using the PAS antibody.

G. Subcellular localisation of GFP-TRIM24 mutant proteins in mouse primary hepatocytes. Scale bars indicate 10 μm in length.

Source data are provided as a Source Data file.

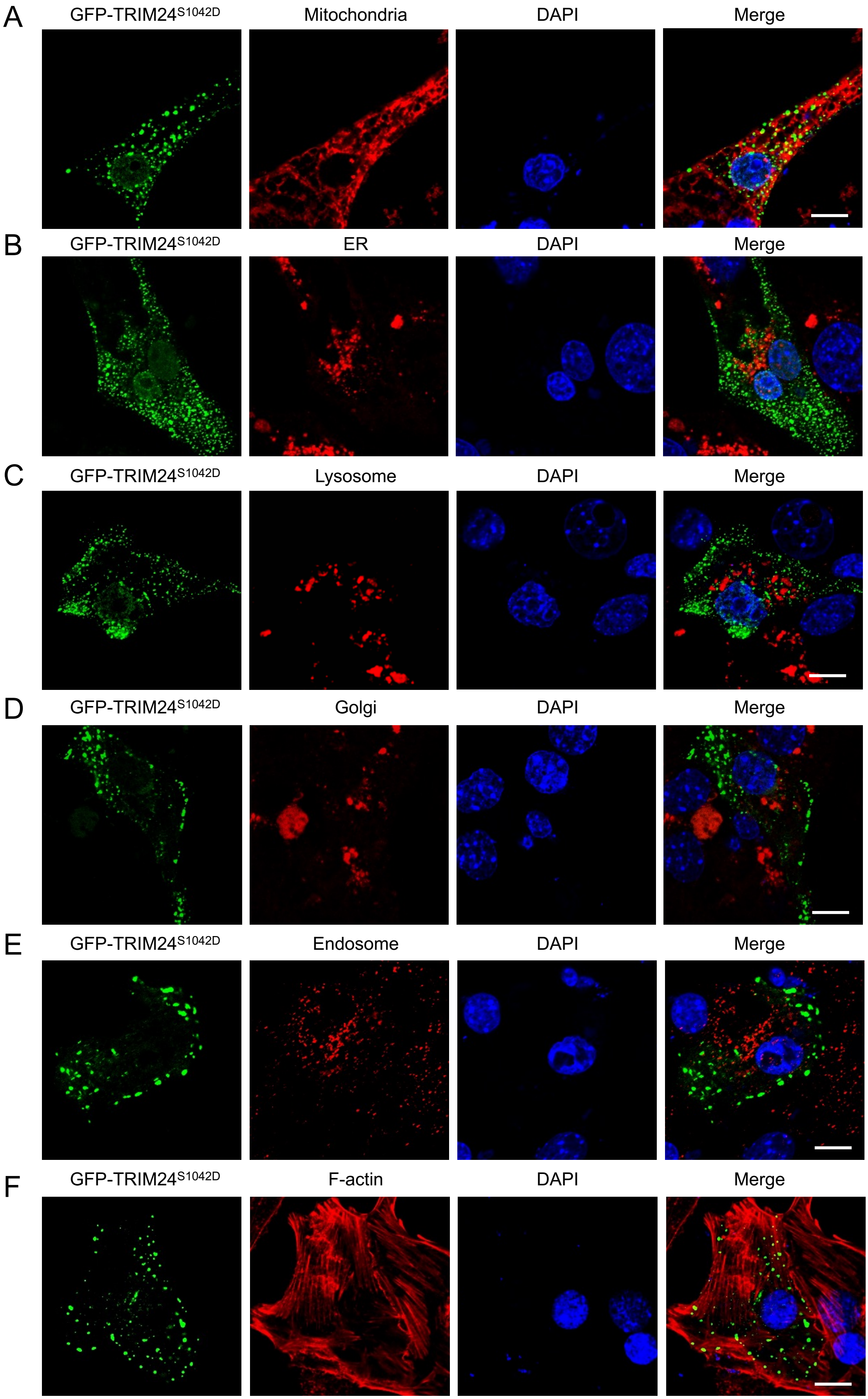

Supplementary Figure 3 Colocalisation of TRIM24 with organelles

GFP-TRIM24^{S1043D} was expressed in mouse primary hepatocytes, and cells were stained with markers for mitochondria (TOM20, A), ER (PDI, B), lysosome (LAMP1, C), Golgi (RCAS1, D), endosome (EEA1, E), and F-actin (F). Nucleus was stained with DAPI. Images of cells were taken using a confocal microscope. Scale bars indicate 10 μm in length.

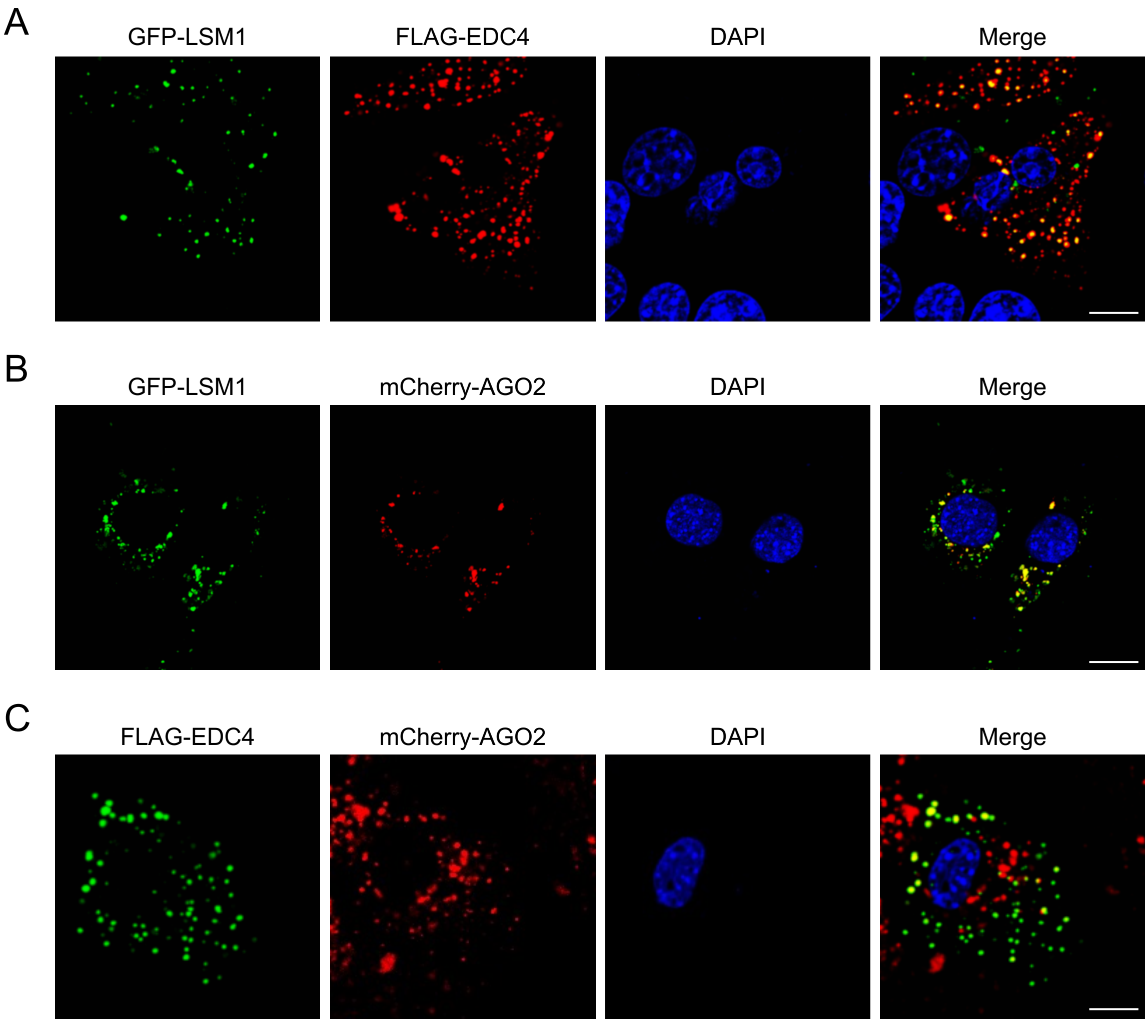

Supplementary Figure 4 Colocalisation of GFP-LSM1, Flag-EDC4 and mCherry-AGO2

A. Colocalisation of GFP-LSM1 and Flag-EDC4. GFP-LSM1 and Flag-EDC4 were co-expressed in primary hepatocytes. After fixation, cells were stained with the Flag antibody and DAPI, and photographed using a confocal microscope. Scale bars indicate 10 μm in length.

B. Colocalisation of GFP-LSM1 and mCherry-AGO2. GFP-LSM1 and mCherry-AGO2 were co-expressed in primary hepatocytes. After fixation, cells were stained with DAPI, and photographed using a confocal microscope. Scale bars indicate 10 μm in length.

C. Colocalisation of Flag-EDC4 and mCherry-AGO2. Flag-EDC4 and mCherry-AGO2 were co-expressed in primary hepatocytes. After fixation, cells were stained with the Flag antibody and DAPI, and photographed using a confocal microscope. Scale bars indicate 10 μm in length.

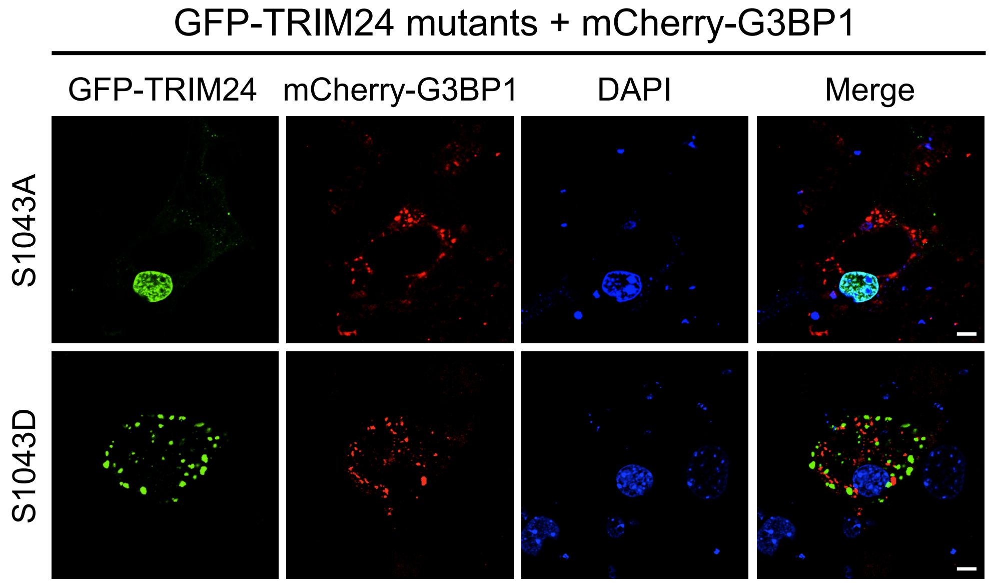

Supplementary Figure 5   Colocalisation of TRIM24 with stress granules
GFP-TRIM24^{S1042A} and GFP-TRIM24^{S1042D} were co-expressed with a stress granule marker mCherry-G3BP1 in mouse primary hepatocytes. After fixation, cells were stained with DAPI, and photographed using a confocal microscope. Scale bars indicate 10 µm in length.

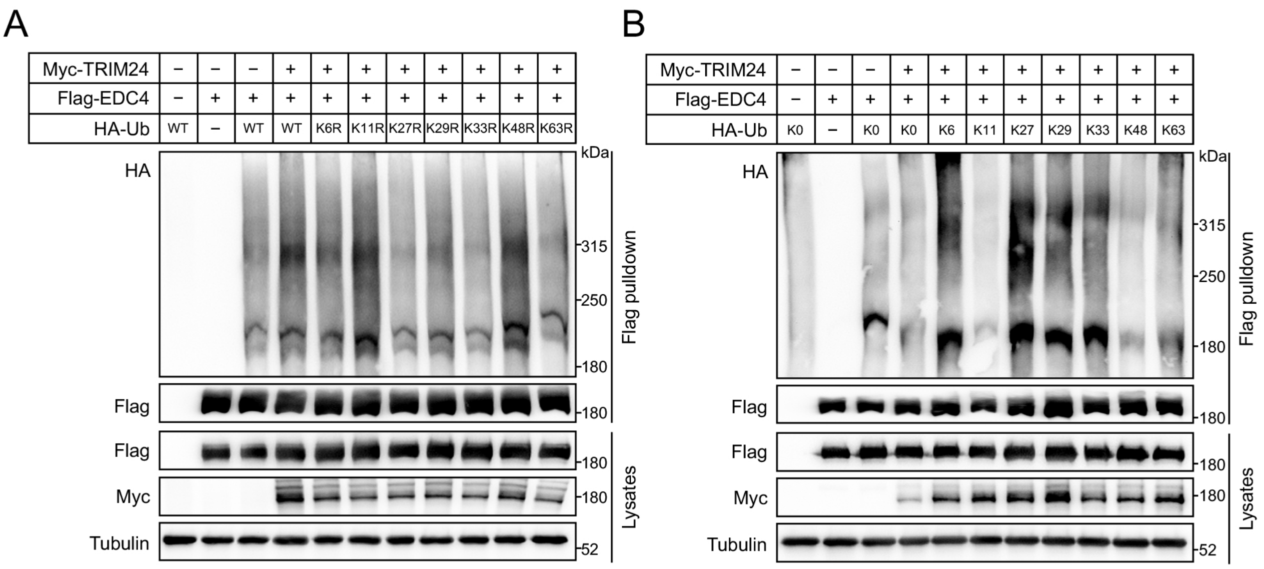

Supplementary Figure 6 Ubiquitination of EDC4 by TRIM24

A. Chain type of polyubiquitination of EDC4 by TRIM24. Flag-EDC4 together with Myc-TRIM24 was co-expressed with HA-Ub WT, K6R, K11R, K27R, K29R, K33R, K48R or K63R in HEK293 cells. After immunoprecipitation, polyubiquitination of Flag-EDC4 was detected via immunoblotting using the HA antibody.

B. Chain type of polyubiquitination of EDC4 by TRIM24. Flag-EDC4 together with Myc-TRIM24 was co-expressed with HA-Ub K0, K6, K11, K27, K29, K33, K48 or K63 only mutants in HEK293 cells. After immunoprecipitation, polyubiquitination of Flag-EDC4 was detected via immunoblotting using the HA antibody.

Source data are provided as a Source Data file.

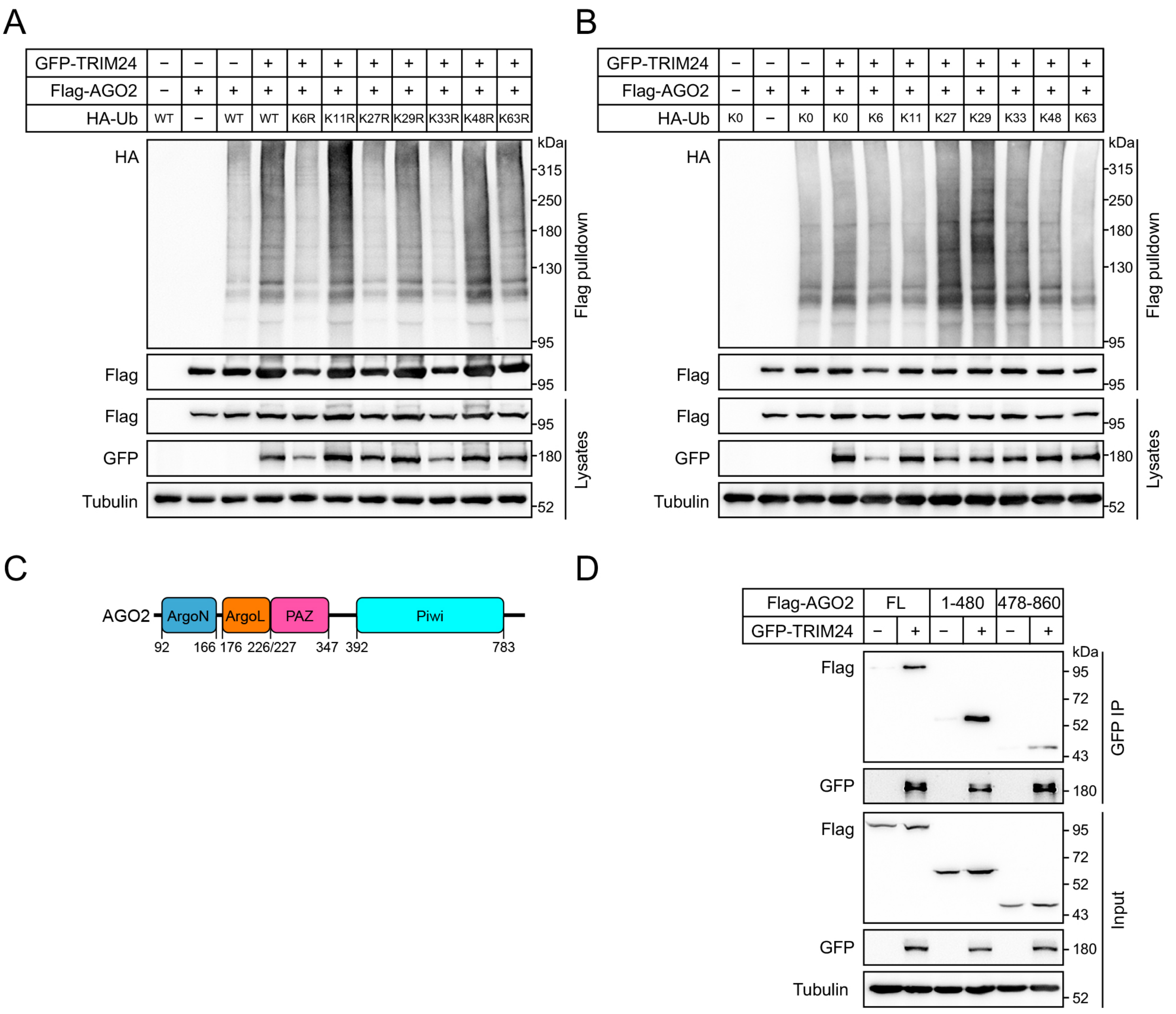

Supplementary Figure 7 Ubiquitination of AGO2 by TRIM24

A. Chain type of polyubiquitination of AGO2 by TRIM24. Flag-AGO2 together with GFP-TRIM24 was co-expressed with HA-Ub WT, K6R, K11R, K27R, K29R, K33R, K48R or K63R in HEK293 cells. After immunoprecipitation, polyubiquitination of Flag-AGO2 was detected via immunoblotting using the HA antibody.

B. Chain type of polyubiquitination of AGO2 by TRIM24. Flag-AGO2 together with GFP-TRIM24 was co-expressed with HA-Ub K0, K6, K11, K27, K29, K33, K48 or K63 only mutants in HEK293 cells. After immunoprecipitation, polyubiquitination of Flag-AGO2 was detected via immunoblotting using the HA antibody.

C. Diagrammatic illustration of domain compositions of AGO2.

D. Mapping of interaction domains of AGO2 with TRIM24. Flag-tagged full-length or domains of AGO2 were co-expressed with GFP-TRIM24 in HEK293 cells. GFP-TRIM24 was immunoprecipitated using the GFP antibody, and Flag-tagged full-length or domains of AGO2 were detected in the immunoprecipitates.

Source data are provided as a Source Data file.

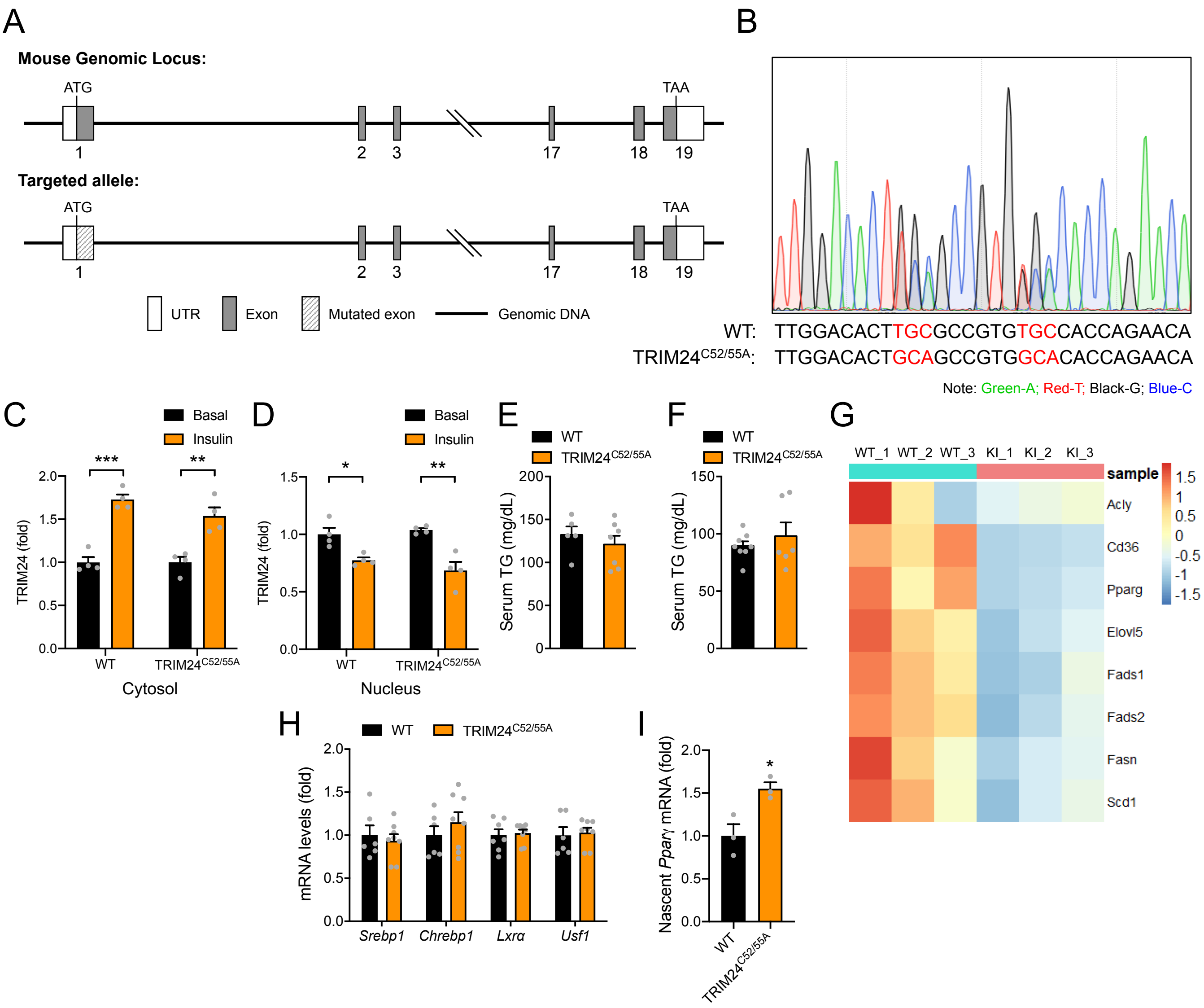

Supplementary Figure 8 Generation and characterization of the TRIM24$^{C52/55A}$ mice

A. Diagrammatic illustration of the strategy for generation of the TRIM24$^{C52/55A}$ mice.

B. Genomic DNA sequences of the WT and TRIM24$^{C52/55A}$ allele.

C-D. Subcellular distribution of endogenous TRIM24 in TRIM24$^{C52/55A}$ and WT hepatocytes in response to insulin. After cellular fractionation, subcellular distribution of TRIM24 was measured in the nuclear and cytosolic fractions via immunoblotting. Representative immunoblots were shown in Fig. 6B. Quantitation of cytosolic TRIM24 (C) and nuclear TRIM24 (D) were shown here. n = 4. C: $p < 0.0001$ (WT/Insulin vs WT/Basal), and $p = 0.0011$ (TRIM24$^{C52/55A}$/Insulin vs TRIM24$^{C52/55A}$/Basal). D: $p = 0.031$ (WT/Insulin vs WT/Basal), and $p = 0.0013$ (TRIM24$^{C52/55A}$/Insulin vs TRIM24$^{C52/55A}$/Basal).

E. Serum TG levels in 5-month-old male TRIM24$^{C52/55A}$ and WT mice fed the CD. n = 5 (WT) and 7 (TRIM24$^{C52/55A}$).

F. Serum TG levels in 5-month-old male TRIM24$^{C52/55A}$ and WT mice fed the HFD. n = 8 (WT) and 6 (TRIM24$^{C52/55A}$).

G. Heat map of lipid metabolic gene expression in the RNA-Seq assay.

H. *Srebp1*, *Chrebp1*, *Lxrα* and *Usf1* mRNA in the liver of 5-month-old male TRIM24$^{C52/55A}$ and WT mice on HFD for 3 months. n = 6 (WT/*Srebp1*, *Chrebp1* and *Usf1*), 7 (WT/*Lxrα*) and 8 (TRIM24$^{C52/55A}$).

I. Nascent *Pparγ* mRNA in primary hepatocytes of TRIM24$^{C52/55A}$ and WT mice. Nucleus were isolated from the liver of TRIM24$^{C52/55A}$ and WT mice, and used for a nuclear run-on assay to measure *Pparγ* mRNA transcription. n = 3. $p = 0.024$.

Data are given as the mean ± SEM. Statistical analyses: two-sided t-test for E, F, H and I, and two-way ANOVA for C and D. * indicates $p < 0.05$, ** $p < 0.01$, and *** $p < 0.001$. Source data are provided as a Source Data file.

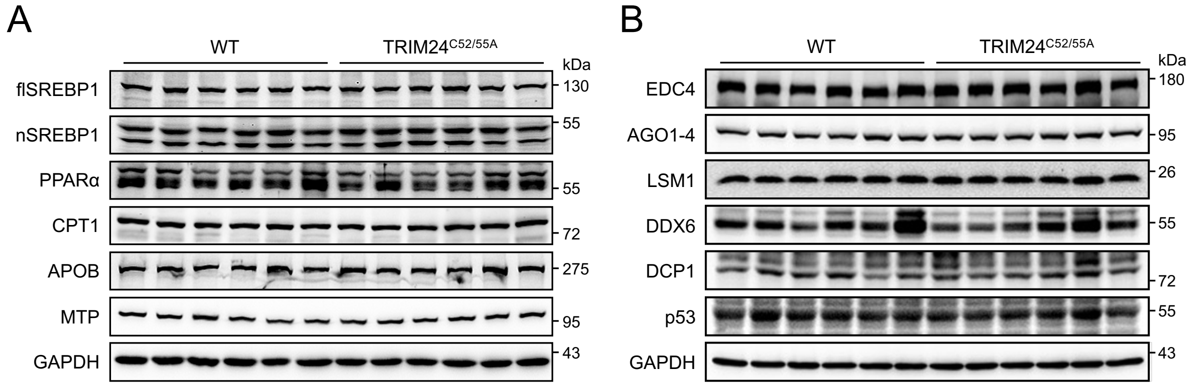

Supplementary Figure 9 Hepatic protein expression in the TRIM24$^{C52/55A}$ mice

A. Protein expression of full-length SREBP1 (flSREBP1), N-terminal SREBP1 (nSREBP1), PPARα, CPT1, APOB and MTP in the liver of 5-month-old male TRIM24$^{C52/55A}$ and WT mice on HFD for 3 months.

B. EDC4, AGO1-4, LSM1, DDX6, DCP1 and p53 in the liver of 5-month-old male TRIM24$^{C52/55A}$ and WT mice on HFD for 3 months.

Source data are provided as a Source Data file.

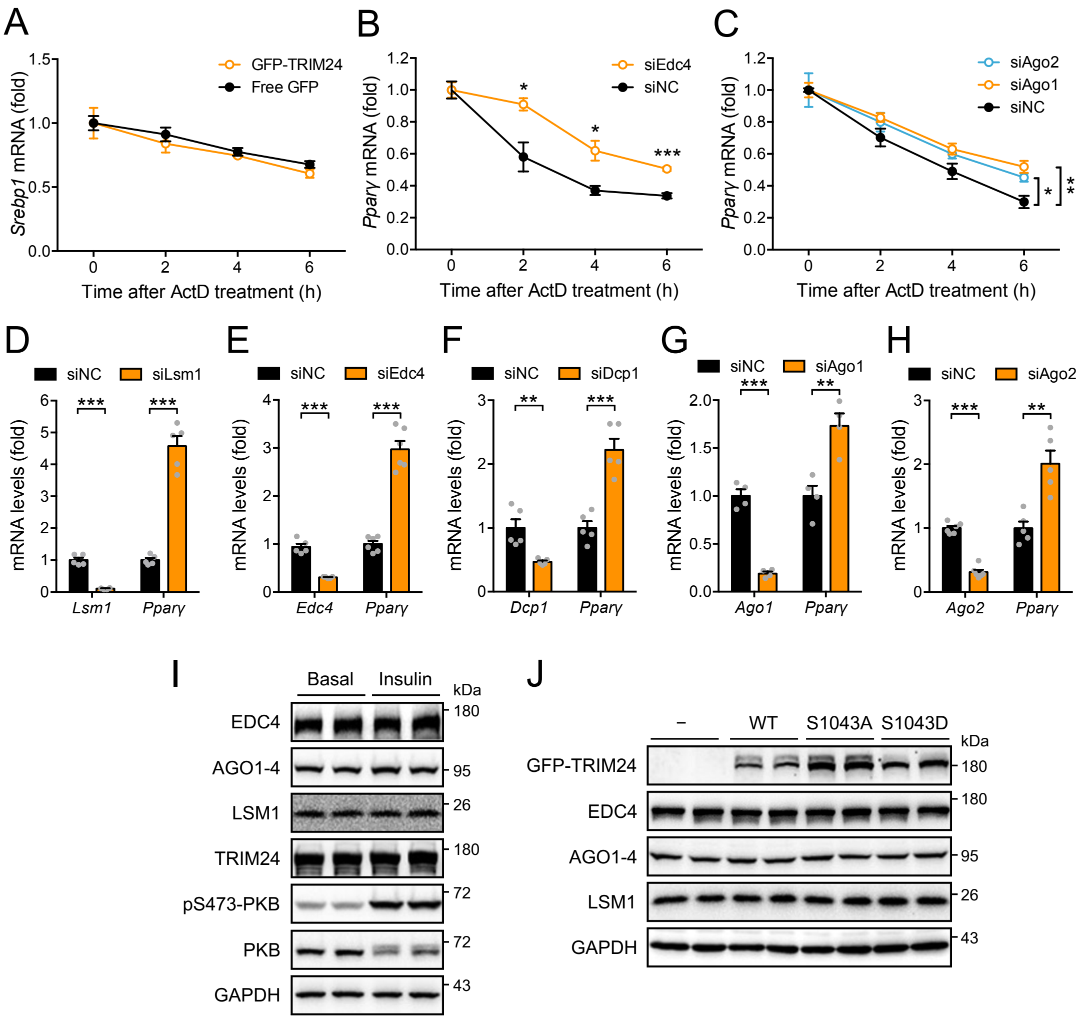

Supplementary Figure 10 Regulation of *Pparγ* mRNA by P-bodies

A. Stability of *Srebp1* mRNA in mouse primary hepatocytes upon overexpression of GFP-TRIM24. Primary hepatocytes were transfected with GFP-TRIM24 or free GFP for 48 h, and then treated with ActD for indicated time before lysis. n = 3.

B-C. Stability of *Pparγ* mRNA in mouse primary hepatocytes upon down-regulation of *Edc4* (B), *Ago1* (C), or *Ago2* (C). Primary hepatocytes were transfected with si*Edc4*, si*Ago1*, si*Ago2*, or siNC for 48 h, and then treated with ActD for indicated time before lysis. n = 4. B, p = 0.016 (siEdc4/2h vs siNC/2h), 0.011 (siEdc4/4h vs siNC/4h) and 0.00017 (siEdc4/6h vs siNC/6h). C, p = 0.0029 (siAgo1/6h vs siNC/6h), 0.032 (siAgo2/6h vs siNC/6h).

D-H. *Pparγ* mRNA levels in mouse primary hepatocytes upon down-regulation of *Lsm1* (D), *Edc4* (E), *Dcp1* (F), *Ago1* (G), or *Ago2* (H). D: n = 5 (siNC/*Lsm1*, siNC/*Pparγ*, siLsm1/*Pparγ*) and 6 (siLsm1/*Lsm1*). p = 3.09e-7 (siNC/*Lsm1* vs siLsm1/*Lsm1*) and 3.58e-6 (siNC/*Pparγ* vs siLsm1/*Pparγ*). E: n = 5 (siNC/*Edc4*, siEdc4/*Edc4*) and 6 (siNC/*Pparγ*, siEdc4/*Pparγ*). p = 1.19e-5 (siNC/*Edc4* vs siEdc4/*Edc4*) and 8.88e-7 (siNC/*Pparγ* vs siEdc4/*Pparγ*). F: n = 5. p = 0.0044 (siNC/*Dcp1* vs siDcp1/*Dcp1*) and 0.00031 (siNC/*Pparγ* vs siDcp1/*Pparγ*). G: n = 4. p = 2.50e-5 (siNC/*Ago1* vs siAgo1/*Ago1*) and 0.0049 (siNC/*Pparγ* vs siAgo1/*Pparγ*). H: n = 6 (siNC/*Ago2*, siAgo2/*Ago2*) and 5 (siNC/*Pparγ*, siAgo2/*Pparγ*). p = 1.19e-7 (siNC/*Ago2* vs siAgo2/*Ago2*) and 0.0022 (siNC/*Pparγ* vs siAgo2/*Pparγ*).

I. Protein expression of EDC4, AGO1-4, LSM1 and TRIM24 in the liver of WT mice that were stimulated with or without insulin.

J. Protein expression of EDC4, AGO1-4 and LSM1 in primary hepatocytes expressing GFP-TRIM24 WT, S1043A, or S1043D mutants.

Data are given as the mean ± SEM. Statistical analyses were carried out via two-sided t-test for A-B, D-H, and via two-way ANOVA for C. * indicates $p < 0.05$, ** indicates $p < 0.01$, and *** indicates $p < 0.001$. Source data are provided as a Source Data file.

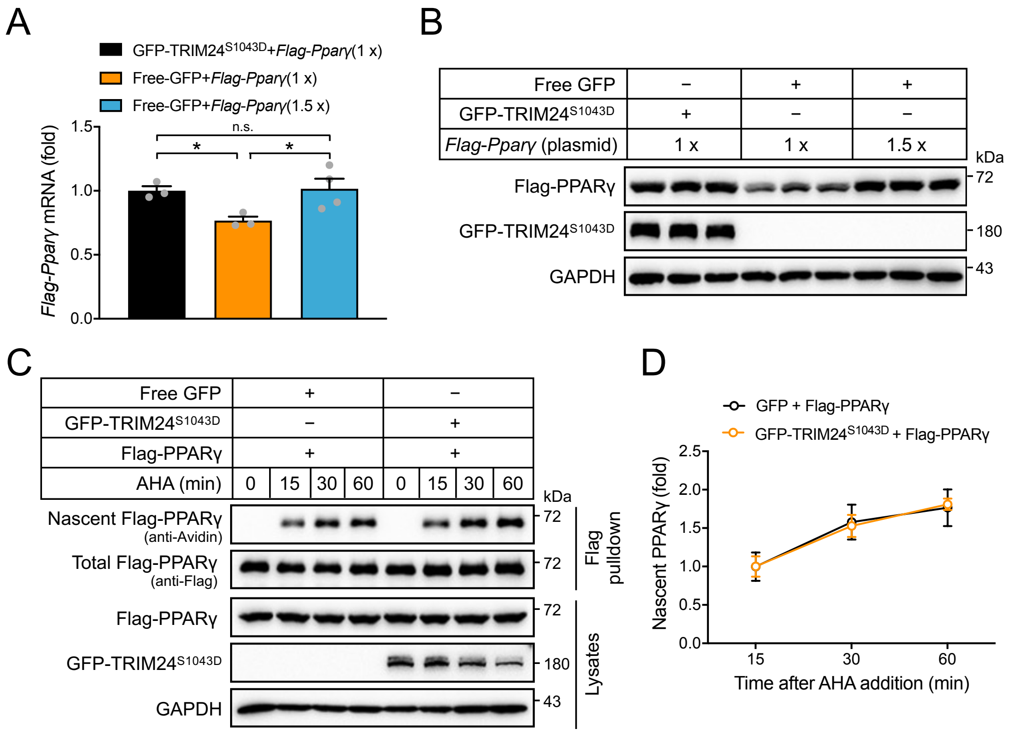

Supplementary Figure 11 Effects of GFP-TRIM24^{S1043D} on the production of nascent Flag-PPARγ

A. mRNA levels of *Flag-Pparγ* in HEK293 cells co-transfected with GFP-TRIM24^{S1043D} or free GFP. For transfection, 1 x *Flag-Pparγ* plasmid means 2 µg plasmid DNA per 6-cm dish, and 1.5 x *Flag-Pparγ* plasmid means 3 µg plasmid DNA per 6-cm dish. n = 3 (GFP-TRIM24^{S1043D}+*Flag-Pparγ*(1x), Free-GFP+*Flag-Pparγ*(1x)) and 4 (Free-GFP+*Flag-Pparγ*(1.5x)). n.s. not significant. p = 0.039 (TRIM24^{S1043D}+*Flag-Pparγ*(1x) vs Free-GFP+*Flag-Pparγ*(1x)) and 0.023 (Free-GFP+*Flag-Pparγ*(1x) vs Free-GFP+*Flag-Pparγ*(1.5x)).

B. Protein levels of Flag-PPARγ in HEK293 cells co-transfected with GFP-TRIM24^{S1043D} or free GFP. For transfection, 1 x *Flag-Pparγ* plasmid means 2 µg plasmid DNA per 6-cm dish, and 1.5 x *Flag-Pparγ* plasmid means 3 µg plasmid DNA per 6-cm dish.

C-D. Production of nascent Flag-PPARγ in HEK293 cells co-transfected with GFP-TRIM24^{S1043D} or free GFP. 1 x *Flag-Pparγ* plasmid (2 µg plasmid DNA per 6-cm dish) was transfected into HEK293 cells co-expressing GFP-TRIM24^{S1043D} while 1.5 x *Flag-Pparγ* plasmid (3 µg plasmid DNA per 6-cm dish) was transfected into HEK293 cells co-expressing free GFP. Two days after transfection, cells were treated with Click-iT^{TM} AHA for the indicated time. Total Flag-PPARγ was immunoprecipitated using the Flag beads, and nascent Flag-PPARγ were subsequently reacted with Click-iT^{TM} Protein Reaction Buffer Kit. The labelled nascent Flag-PPARγ was then detected using HRP-labelled Avidin antibody. C, representative blots. D, quantification of nascent Flag-PPARγ. n = 5 for GFP-TRIM24^{S1043D}+Flag-PPARγ/60 min, and n = 6 for the rest.

Data are given as the mean ± SEM. Statistical analyses were carried out via one-way ANOVA for A and via two-sided t-test for D. * indicates $p < 0.05$. Source data are provided as a Source Data file.

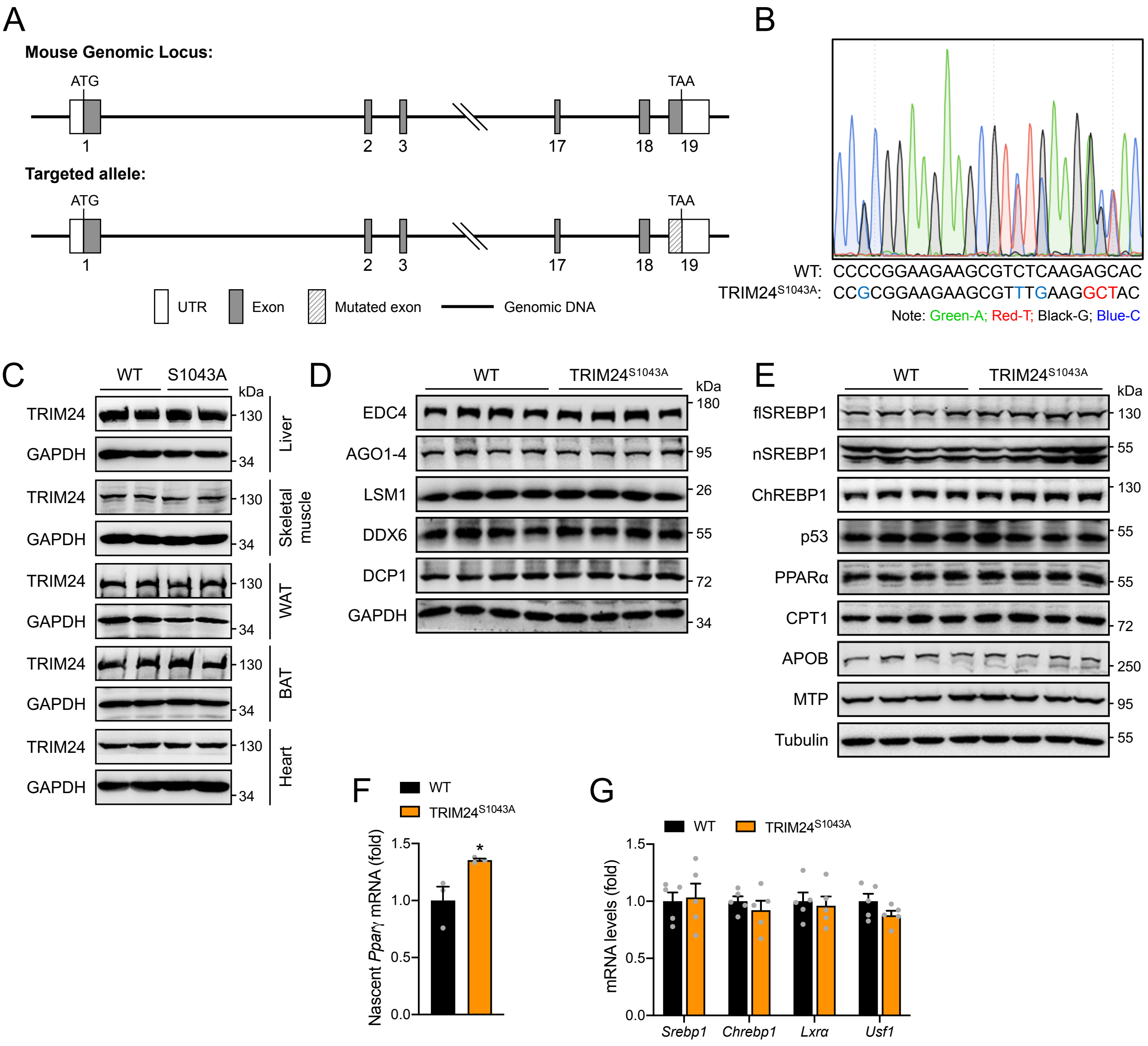

Supplementary Figure 12 Generation and characterization of the TRIM24^{S1043A} mice

A. Diagrammatic illustration of the strategy for generation of the TRIM24^{S1043A} mice.

B. Genomic DNA sequences of the WT and TRIM24^{S1043A} allele.

C. Expression of TRIM24 protein in various tissues of 6-month-old male TRIM24^{S1043A} and WT mice on CD.

D. Protein expression of EDC4, AGO1-4, LSM1, DDX6 and DCP1 in the liver of 6-month-old male TRIM24^{S1043A} and WT mice on HFD.

E. Protein expression of full-length SREBP1 (flSREBP1), N-terminal SREBP1 (nSREBP1), ChREBP1, p53, PPARα, CPT1, APOB and MTP in the liver of 6-month-old male TRIM24^{S1043A} and WT mice on HFD.

F. Nascent *Pparγ* mRNA in primary hepatocytes of TRIM24^{S1043A} and WT mice. Nucleus were isolated from the liver of TRIM24^{S1043A} and WT mice, and used for a nuclear run-on assay to measure *Pparγ* mRNA transcription. n = 3. $p = 0.044$.

G. mRNA levels of *Srebp1*, *Chrebp1*, *Lxrα* and *Usf1* in the liver of 6-month-old male TRIM24^{S1043A} and WT mice on HFD for 4 months. n = 5.

Data are given as the mean ± SEM. Statistical analysis was carried out via two-sided t-test for F-G. * indicates $p < 0.05$. Source data are provided as a Source Data file.
