# Supplementary material for: TRIM24 is an insulin-responsive regulator of P-bodies
Source: Nat Commun. 2022 Jul 8;13:3972. doi: 10.1038/s41467-022-31735-0 (PMC9270398; doi:10.1038/s41467-022-31735-0)
Supplement: Supplementary file 2 — Description of Additional Supplementary Files [file 41467_2022_31735_MOESM2_ESM.pdf]

Description of Additional Supplementary Files

Supplementary Data 1 Proteins identified in the PAS immunoprecipitates from mouse liver lysates

PAS-reactive phosphorylated proteins were immunoprecipitated from mouse liver lysates using the PAS antibody. The immunoprecipitated proteins were electrophoretically separated via SDS-PAGE, and stained with Coomassie blue dye. Immunoprecipitated proteins were excised and subjected to identification via mass-spectrometry. Proteins that had at least two unique peptides identified were considered as positive hits.

Supplementary Data 2 P-body components identified in the immunoprecipitates of GFP-TRIM24

GFP-TRIM24 was expressed in HEK293 cells, and immunoprecipited from cell lysates using the GFP-Trap®-agarose. Immunoprecipitates were separated via SDS-PAGE, stained with Coomassie blue dye, and subjected to identification via mass-spectrometry. Proteins that had at least two unique peptides identified were considered as positive hits. P-body components identified in the GFP-TRIM24 immunoprecipitates are listed.

Supplementary Data 3 Differentially-expressed genes in the liver of HFD-fed TRIM24$^{C52/55A}$ mice

Deep sequencing of RNA (RNA-Seq) was performed in the liver of HFD-fed WT and TRIM24$^{C52/55A}$ mice. Genes with differential expression are listed in this table.
